# Mosaic chromosome Y loss is associated with alterations in blood cell counts in UK Biobank men

**DOI:** 10.1038/s41598-020-59963-8

**Published:** 2020-02-27

**Authors:** Shu-Hong Lin, Erikka Loftfield, Josh N. Sampson, Weiyin Zhou, Meredith Yeager, Neal D. Freedman, Stephen J. Chanock, Mitchell J. Machiela

**Affiliations:** 1 Division of Cancer Epidemiology and Genetics, National Cancer Institute, Rockville, 9609 Medical Center Drive MSC 9776, Bethesda, Maryland 20892 USA; 2 Cancer Genomics Research Laboratory, Frederick National Laboratory for Cancer Research, Frederick, Maryland, 8717 Grovemont Circle, Gaithersburg, MD 20877 USA

**Keywords:** Genetics, Biomarkers, Risk factors

## Abstract

Mosaic loss of Y chromosome (mLOY) is the most frequently detected somatic copy number alteration in leukocytes of men. In this study, we investigate blood cell counts as a potential mechanism linking mLOY to disease risk in 206,353 UK males. Associations between mLOY, detected by genotyping arrays, and blood cell counts were assessed by multivariable linear models adjusted for relevant risk factors. Among the participants, mLOY was detected in 39,809 men. We observed associations between mLOY and reduced erythrocyte count (−0.009 [−0.014, −0.005] × 10^12^ cells/L, *p* = 2.75 × 10^−5^) and elevated thrombocyte count (5.523 [4.862, 6.183] × 10^9^ cells/L, *p* = 2.32 × 10^−60^) and leukocyte count (0.218 [0.198, 0.239] × 10^9^ cells/L, *p* = 9.22 × 10^−95^), particularly for neutrophil count (0.174 × [0.158, 0.190]10^9^ cells/L, *p* = 1.24 × 10^−99^) and monocyte count (0.021 [0.018 to 0.024] × 10^9^ cells/L, *p* = 6.93 × 10^−57^), but lymphocyte count was less consistent (0.016 [0.007, 0.025] × 10^9^ cells/L, *p* = 8.52 × 10^−4^). Stratified analyses indicate these associations are independent of the effects of aging and smoking. Our findings provide population-based evidence for associations between mLOY and blood cell counts that should stimulate investigation of the underlying biological mechanisms linking mLOY to cancer and chronic disease risk.

## Introduction

Recently, large molecular epidemiology studies have shown that hematopoietic cells can undergo postzygotic mutations resulting in somatic copy number alterations, which can give rise to daughter cells with the same genomic aberration. Clonal expansion of cells bearing a somatic mutation results in clonal mosaicism^1^. Clonal mosaicism can be driven by somatic mutations affecting genes frequently mutated in myelodysplastic disease (referred to as clonal hematopoiesis of indeterminate potential (CHIP))^2^ or be the result of acquired copy number aberrations. The most frequently detected somatic copy number alteration in circulating leukocytes is mosaic loss of the Y chromosome (mLOY) in males^3–5^. The prevalence of mLOY is age-related, increasing substantially after age 50 in men. Likewise, CHIP is common in the elderly, with recent evidence suggesting CHIP and mLOY may co-occur^6^. Exposure to cigarette smoking^4,7^ has also been well established as a risk factor for mLOY and recently, early evidence suggests that air pollution could also be associated with mLOY^8^. Epidemiologic studies have uncovered potential associations between mLOY and increased risk of cancer^3–5^, neurodegenerative diseases^9^, and cardiovascular diseases^10,11^. Similarly, CHIP has been associated with select cancers and cardiovascular disease^2^. Although common germline variants near important cell-cycle regulation and cancer susceptibility genes have been identified through genome-wide association studies of mLOY^4,12^, the underlying biologic mechanisms linking mLOY to chronic disease risk are likely complex.

We investigated possible associations between mLOY and clinical measures, here the composition of the classical hematologic compartments in the UK Biobank, which has available genomic and blood cell count data on over 220,000 men, providing a large well-characterized population to investigate these questions. We performed multivariable, stratified and mediation analyses to evaluate the association of mLOY with blood cell count and distribution. Our study provides a unique, population-based investigation of changes in blood cell counts associated with a somatic mutation. Our findings indicate robust associations between mLOY and leukocyte, erythrocyte and thrombocyte counts, independent of age and cigarette smoking.

## Methods

The current analyses were extension of our previous studies^5,13,14^ which included population-based data from 223,336 males between age 37 and 73 recruited between 2007 to 2010 from the UK Biobank^15,16^. After providing informed consent, each participant provided a blood sample, answered a detailed health and lifestyle questionnaire, and had physical measurements taken. Collected blood was held at 4 °C and sent to a central processing laboratory in temperature-controlled boxes. Samples were processed, aliquoted and cryopreserved at −80 °C or −196 °C. One tube of blood was loaded on a Beckman Coulter LH750 to provide direct measurement of various blood cell counts and indices including but not limiting to hematocrit, plateletcrit, mean corpuscular volume, hemoglobin, mean corpuscular hemoglobin, mean corpuscular hemoglobin concentration, mean sphered cell volume, mean platelet volume, platelet distribution width, immature reticulocyte fraction, and high light scatter reticulocyte proportion.

Blood-derived DNA from UK Biobank men was extracted starting early 2013 and genotyped on Affymetrix UK BiLEVE or UK Biobank Axiom arrays. mLOY was measured by two different methods. The first measure of mLOY was made by examining the median log R ratio (mLRR) of 691 single nucleotide polymorphisms spanning the male specific region of the Y chromosome. LRR is a measure of probe signal intensity on the genotyping array with negative values across contiguous variants indicating evidence for a copy number loss and positive values indicating copy number gain. Men with mLRR > 0.15 could possibly have a mosaic gain of Y chromosome or a constitutional extra copy of the Y chromosome (XYY syndrome) and were removed (205 men, 0.09%). We also apply the methods proposed by Thompson *et al*.^14^ to call dichotomized mLOY. For clarity, we use the term mLOY for dichotomized mLOY calls by Thompson *et al*.

**Figure 1:**
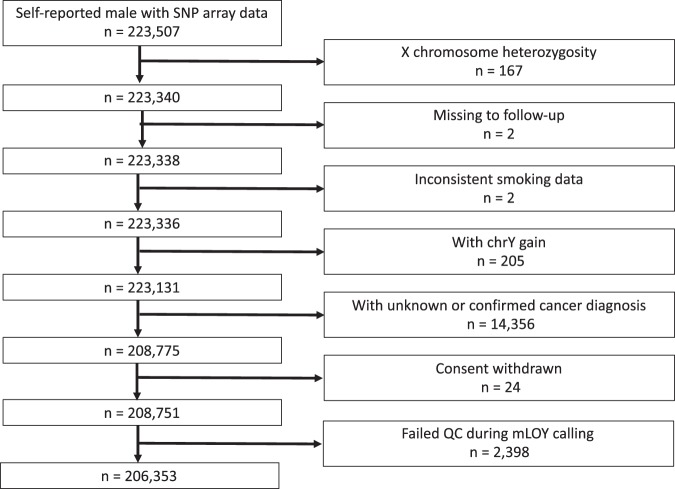
Number of participants. The number of participants excluded by each criterion was shown.

Our final analytical set included 206,353 self-reported men who reported no prior cancer history at recruitment (n = 14,356 excluded), did not have X chromosome heterozygosity (n = 167 excluded), had consistent smoking data (n = 2 excluded), were without evidence of copy number gains of Y chromosome (n = 205 excluded) and passed quality control during the dichotomized mLOY detection step (n = 2,394 excluded) as shown in Fig. 1. Multivariable linear regression was employed to identify associations between lifestyle factors, mLOY and blood cell counts. Factors which might influence blood cell counts (smoking^17^, BMI^18^, alcohol consumption^19^, diabetes^20^, hypertension^21^, and hypoercholesterolemia^22^) were included in multivariable models. For interaction between smoking and mLOY, we fit models with mLRR, 3-level smoking status (never, former, current), interaction terms between mLRR and smoking, and all other aforementioned covariates. We adjusted for continuous age, age squared^13^, race/ethnicity, smoking, alcohol consumption, continuous body mass index (BMI), self-reported diabetes, hypertension, and hypercholesterolemia. When modeling mLRR, we applied the following formula to calculate standardized mLRR for easier interpretation of the regression coefficient. We first subtracted mean of raw mLRR (mLRR_r_) from all raw mLRR, divided the difference by standard deviation (S.D.) of mLRR_r_, and flipped the sign (to have effect estimates in the same direction as the dichotomous mLOY estimates).

$${\rm{mLRR}}=-\,\frac{{{\rm{mLRR}}}_{{\rm{r}}}-\overline{{{\rm{mLRR}}}_{{\rm{r}}}}}{{\rm{S}}.{\rm{D}}.({{\rm{mLRR}}}_{{\rm{r}}})}$$

A 25-level smoking status variable was created according to prior literature^13^ and used in the majority of our models. In brief, a detailed smoking history variable was created by combining information on baseline smoking status, smoking intensity, time since quitting, and type of tobacco product smoked (i.e., cigarettes or cigars/pipes). Indicator variables were created for each category and “never smokers” were used as the reference group. After accounting for skip patterns, a small percentage of respondents were missing one or more pieces of information. Because we had some, but not complete, smoking information on these respondents we created a number of indicator variables for each of the partial missing categories. Alcohol consumption was coded in 7 levels (never, former, occasional, 1–3 drink/month, 1–2 drink/week, 3–4 drink/week) according to the history and frequency of consumption. Continuous BMI was used in most models while a 5-level BMI defined by the World Health Organization (WHO) was also presented for reference. All missing data in categorical variables were coded as ‘missing’ and included in the analysis. A significance level was set at 0.001 to account for multiple comparisons (0.05/50 independent tests for all indicator variables). To evaluate the effect of any potential residual confounding from smoking and age in the reported association between mLOY and blood cell counts, we conducted a sensitivity analysis restricted to strata of ever or never smokers and individuals older or younger than 65 years old. We chose 65 years old because the prevalence of mLOY increased exponentially between 60 to 70 years old even for never smokers (Fig. S1).

A polygenic risk score (PRS) was created to estimate the effect of genetic contributors to mLOY. The PRS was calculated by scoring risk alleles in 156 variants previously found to be associated with mLOY risk using the formula:

$${{\rm{PRS}}}_{{\rm{i}}}=\mathop{\sum }\limits_{{\rm{j}}=1}^{156}{{\rm{\beta }}}_{{\rm{i}}}({{\rm{SNP}}}_{{\rm{ij}}})$$

*i* denotes an individual while *j* denotes a SNP being scored. The number of risk alleles of each variant (SNP_*ij*_) were weighted by reported estimates for log odds ratio of risk alleles (β)^23^. Then, blood cell counts and other indices were regressed against the PRS instrumental variable to investigate for potential associations between mLOY PRS and blood counts. Mediation analyses were conducted to estimate the direct and indirect effect among smoking, mLOY, and blood cell counts while controlling for BMI, ethnicity, alcohol consumption, diabetes, hypertension and hypercholesterolemia using the “mediation” package in R^24^.

Finally, we performed reciprocal Mendelian randomization (MR) on previously reported mLOY-associated SNPs as well as blood cell count-associated SNPs obtained from PhenoScanner^25,26^. We queried for respective blood cell counts in PhenoScanner and discarded multi-allelic SNPs. The final list of SNPs included 270 for leukocyte count^27–39^, 1,888 for erythrocyte count^29,31–36,39–48^, and 662 for thrombocyte count^29,31–36,44,49–55^. Estimation of causal association was carried out by the “MendelianRandomization” package^56^. All statistical analyses were performed in R version 3.5.2 and plots were created using the “ggplot2”^57^ and “sjPlot”^58^ packages.

### Ethical approval

The UK Biobank received ethical approval from the research ethics committee (REC reference for UK Biobank 21552) and all participants provided signed informed consent at enrollment and all research was performed in accordance with relevant guidelines/regulations. All data used in this analysis is available through application to the UK Biobank.

## Results

**Figure 2:**
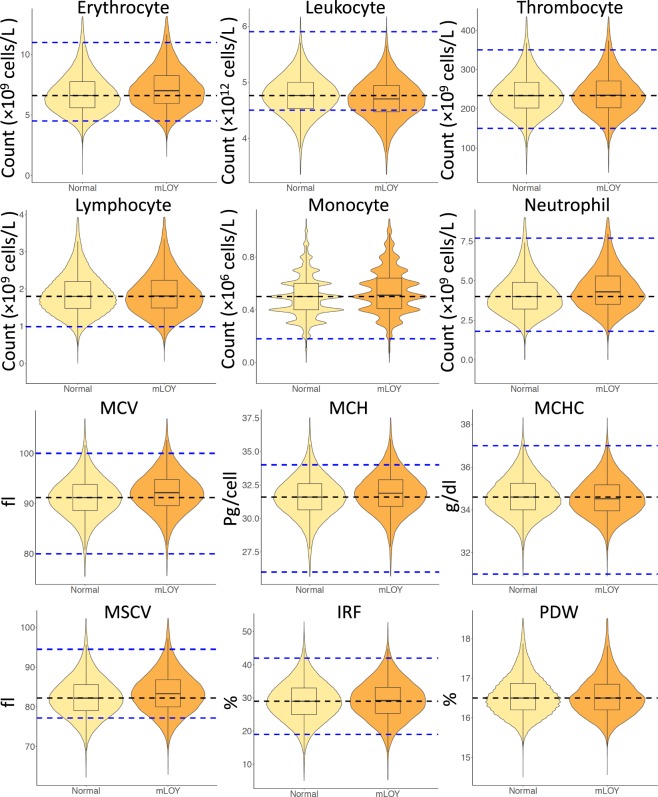
Blood cell counts and indices by mLOY status. Various blood cell counts among men with different mLOY status were observed in univariate analyses. Yellow, Normal: men with normal chromosome Y; Orange, mLOY: men with mLOY. All displayed counts and indices had *p* < 0.001 when comparing between participants with or without mLOY. Black dashed lines: median count among normal individuals. Blue dashed lines: reference ranges from prior studies^73–75^. MCV: mean corpuscular volume. MCH: mean corpuscular hemoglobin. MCHC: mean corpuscular hemoglobin concentration. MSCV: mean sphered corpuscular volume. IRF: immature reticulocyte fraction. PDW: platelet distribution width.

Patient characteristics are shown in Table 1 based on their mLOY status. Among the 206,353 UK Biobank male subjects in our analytic set, we detected mLOY in 39,809 men (19.29%). Compared to participants without mLOY, those with mLOY were older (6.683 [6.598, 6.768] years, *p* < 5 × 10^−324^), more likely to be white (OR = 2.822 [2.560, 3.118], 4.014 [3.464, 4.682] compared to Asian and Black, respectively. *p* < 5 × 10^−324^ for both), more likely to smoke (OR = 1.588 [1.551, 1.627] and 1.973 [1.910, 2.038] for former and current compared to never smokers, respectively. *p* < 5 × 10^−324^ for both), more likely to consume alcohol (OR = 1.451 [1.324, 1.591] and 1.326 [1.234, 1.426] for former and current compared to never drinkers, *p* = 8.88 × 10^−16^ and 4.00 × 10^−15^, respectively), less likely to have BMI > 35 (OR = 0.645 [0.513, 0.820], *p* < 5 × 10^−324^), more likely to have diabetes (OR = 1.121 [1.072, 1.171], *p* = 6.71 × 10^−7^), hypertension (OR = 1.344 [1.314, 1.376], *p* < 5 × 10^−324^), and hypercholesterolemia (OR = 1.509 [1.468, 1.552]], *p* < 5 × 10^−324^). As expected, as individuals age, the prevalence of mLOY increased exponentially (Fig. S1). There was no significant difference for mLOY prevalence by assessment center and region (Table S1). In univariable analyses, we detected associations (p < 0.001) between mLOY and counts of six blood cell populations including leukocytes, erythrocytes, thrombocytes, lymphocytes, monocytes, and neutrophils (Fig. 2). We examined possible confounding by immune-related diseases identified in inpatient records and did not identify any statistically significant association between these diseases and mLOY (Table S2).

**Table 1:** Patient characteristics by mLOY status.

Characteristics	Normal	mLOY	*P*^*a*^
**Participants**	166548 (80.7)	39809 (19.3)	NA
**Age (mean,SD)**	55.199 (8.168)	61.882 (5.784)	<5 × 10^−324^
**Ethnicity**			
White	155443 (80)	38790 (20)	Ref
Mixed	922 (90.5)	97 (9.5)	<5 × 10^−324^
Asian	4932 (91.9)	436 (8.1)	<5 × 10^−324^
Black	2914 (94.2)	181 (5.8)	<5 × 10^−324^
Other	1648 (92.3)	138 (7.7)	<5 × 10^−324^
**Smoking status**			
Never	86007 (84.8)	15474 (15.2)	Ref
Former	60851 (77.8)	17388 (22.2)	<5 × 10^−324^
Current	19072 (73.8)	6770 (26.2)	<5 × 10^−324^
**Alcohol drinking**			
Never	4877 (84.7)	883 (15.3)	Ref
Former	5660 (79.2)	1487 (20.8)	8.88 × 10^−16^
Current	155837 (80.6)	37408 (19.4)	4.00 × 10^−15^
**Body mass index**			
18.5-	339 (78.3)	94 (21.7)	0.279
18.5to25	39733 (80.4)	9702 (19.6)	Ref
25to30	80774 (80.3)	19853 (19.7)	0.636
30to35	32845 (81.4)	7507 (18.6)	1.08 × 10^−4^
35+	9864 (84.8)	1767 (15.2)	<5 × 10^−324^
**Diseases**			
Diabetes	9870 (79)	2625 (21)	6.71 × 10^−7^
Hypertension	48007 (77.4)	14033 (22.6)	<5 × 10^−324^
Hypercholesterolemia	24396 (74.9)	8192 (25.1)	<5 × 10^−324^

Abbreviations: mLOY: mosaic loss of the Y chromosome.

^a^Fisher’s exact test with mid-*p* method.

**Figure 3:**
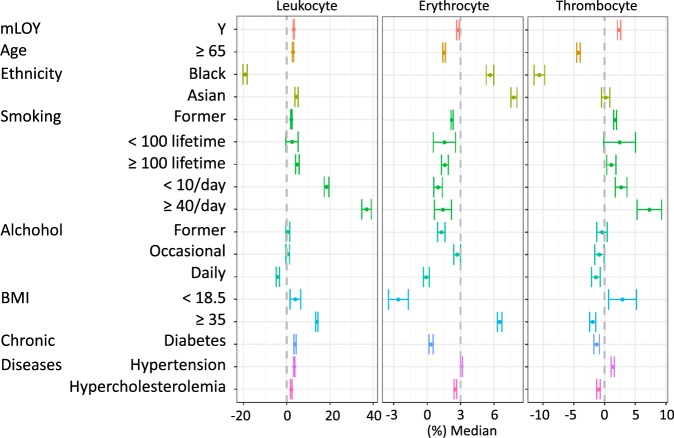
Relative impact of selected risk factors associated with leukocyte, erythrocyte, and thrombocyte counts. Multivariable linear regression models adjusted for age, age squared, race/ethnicity, smoking, alcohol consumption, body mass index (continuous variable), diabetes, hypertension, and hypercholesterolemia. The reference group for categorical variables were no mLOY, Caucasian, never smoker, never drinker, 18.5 ≤ body mass index <25, no diabetes, no hypertension, no hypercholesterolemia. The point estimates, confidence interval, and *p*-values can be found in Table S1.

Multivariable analyses further confirmed this relationship between mLOY and blood cell count for erythrocytes (OR_mLOY_ = −0.009 [−0.014, −0.005] × 10^12^ cells/L, *p*_mLOY_ = 2.75 × 10^−5^; β_mLRR_ = −0.009 [−0.010, −0.007] × 10^12^ cells/L, *p*_mLRR_ = 8.73 × 10^−23^ for categorical mLOY and increase in each standard deviation of mLRR, respectively with all following estimates, 95% CIs, and p values reported in the same order), leukocytes (OR_mLOY_ = 0.218 [0.198, 0.239] × 10^9^ cells/L, *p*_mLOY_ = 9.22 × 10^−95^; β_mLRR_ = 0.058 [0.050 to 0.066] × 10^9^ cells/L, *p*_mLRR_ = 6.48 × 10^−45^) and thrombocytes (OR_mLOY_ = 5.523 [4.862, 6.183] × 10^9^ cells/L, *p*_mLOY_ = 2.32 × 10^−60^; β_mLRR_ = 2.321 [2.063, 2.579] × 10^9^ cells/L, *p* = 2.41 × 10^−69^) (Fig. 3; Table S3). Among these blood cell count associations, mLOY was positively associated in all cases except for erythrocyte count where an inverse association was observed. For leukocyte populations, we observed elevated monocyte (OR_mLOY_ = 0.021 [0.018, 0.024] × 10^9^ cells/L, *p*_mLOY_ = 6.93 × 10^−57^; β_mLRR_ = 0.005 [0.004, 0.006] × 10^9^ cells/L, *p*_mLRR_ = 5.24 × 10^−25^) and neutrophil (OR_mLOY_ = 0.174 [0.158, 0.190] × 10^9^ cells/L, *p*_mLOY_ = 1.24 × 10^−99^; β_mLRR_ = 0.055 [0.048, 0.061] × 10^9^ cells/L, *p*_mLRR_ = 4.81 × 10^−65^) counts associated with mLOY (Table S4). We found inconsistent evidence for an overall association between mLOY and the overall lymphocyte count (OR_mLOY_ = 0.016 [0.007, 0.025] × 10^9^ cells/L, *p*_mLOY_ = 8.52 × 10^−4^; β_mLRR_ = −0.002 [−0.005, 0.002] × 10^9^ cells/L, *p*_mLRR_ = 0.345). While certain immune-related diseases were associated with blood cell counts (Tables S5 and S6), these diseases occurred in less than 1% of individuals and inclusion of these diseases did not significantly alter the estimates for mLOY and other risk factors. All observed blood count associations demonstrated a dose-response relationship which is evident in the mLRR results. The most prominent association was observed for neutrophil count in which men with mLOY had 1.74 × 10^8^ more cells per liter compared to men without detectable mLOY. This is equivalent to an average increase of 4.153% in median neutrophil count among men without mLOY. As a comparison, among erythrocytes, the least associated blood cells, men with mLOY had 9 × 10^9^ fewer erythrocyte per liter compared to men without mLOY, a decrease equivalent to 0.189% of the mean erythrocyte count in individuals without detectable mLOY.

In addition to mLOY, the multivariable model also provided estimates for other potential factors associated with blood cell counts. Smokers who smoke >40 cigarettes per day had leukocyte count 36.91% higher than the median leukocyte count of never smokers with the elevation being evenly distributed across lymphocytes, monocytes, and neutrophils (Figs. 3 and S2). Black ethnicity was associated with 19.26% less leukocyte and 10.61% less thrombocyte compared to Whites, and the decrease in leukocyte count was more prominent among monocytes (19.80%) and neutrophils (32.67%), but relatively mild in lymphocytes (8.56%) suggesting a preferential activation of innate immunity. Alcohol consumption, even among daily drinkers, is mildly associated with blood cell counts, and the largest effect size was observed for decrease in leukocyte count (4.14%) when compared to never drinkers. Another factor associated with blood cell counts is body mass index (BMI) where having BMI greater than 35 was associated with increased leukocyte (13.60%), erythrocyte (2.92%), lymphocyte (16.61%), monocyte (16.86%), and neutrophil (11.72%) counts as well as decreased thrombocyte count (1.91%) compared to those with normal BMI (18.5 to 25).

As indicated in Table S7, neutrophil-lymphocyte ratio (NLR), a measure used to evaluate systemic inflammation in cancer patients, was found to be significantly elevated in men with mLOY (OR_mLOY_ = 0.061 [0.045, 0.077], *p*_mLOY_ = 2.98 × 10^−14^). The thrombocyte-lymphocyte ratio (TLR), a prognostic indicator for select cancer types^59–62^, was also found to be elevated in men with mLOY (OR_mLOY_ = 1.288 [0.564, 2.012], *p*_mLOY_ = 4.90 × 10^−4^). These mLOY associations with NLR and TLR showed robust dose response relationships and demonstrated significant trends with continuous mLRR (*p*_mLRR_ = 1.39 × 10^−33^ and 3.31 × 10^−32^, respectively), suggesting robust relationships for mLOY with measures of both systematic inflammation and cancer prognosis.

To better understand the changes in erythrocyte and platelet counts, we also explored associations between mLOY and multiple erythrocyte indices. Both univariable (Fig. 2) and multivariable (Tables S8 and S9) regression on erythrocyte-related indices indicated that mLOY was associated with an increase in mean corpuscular hemoglobin (MCH), mean corpuscular volume (MCV), immature reticulocyte fraction (IRF), mean sphered cell volume (MSCV), and high light scatter reticulocyte percentage (HLSRP) as well as decrease in hemoglobin (Hb).

To evaluate the potential effect of possible residual confounding from smoking, we conducted a sensitivity analysis restricted to strata of ever or never smokers. For leukocyte, erythrocyte, thrombocyte, monocyte, and neutrophil count, associations with mLOY and cell count remained significant, but the effect size was larger in ever smokers for all counts except thrombocyte count. (Tables S10–S13). Interestingly, lymphocyte count was only significantly increased in ever smokers. We further tested for interactions between mLOY and smoking on changes in blood cell count. mLRR was found to significantly interact with current smoking status in analyses of leukocyte (*p* = 5.06 × 10^−31^), thrombocyte (*p* = 4.18 × 10^−6^), lymphocyte (*p* = 5.25 × 10^−11^), and neutrophil (*p* = 2.26 × 10^−25^) count. Likewise, significant interactions between mLRR and former smoking status were also seen for leukocyte (*p* = 5.30 × 10^−8^) and neutrophil (*p* = 2.33 × 10^−6^) count. Inclusion of interaction terms modified the magnitude of association between mLRR and blood cell counts, but the direction of association between mLRR and cell counts remained the same except for lymphocyte count where the direction of association flipped in never smokers (Fig. S3). The associations between mLOY and NLR remained statically significant in both ever and never smokers (Table S14). We observed a stronger association between TLR and mLOY in never smokers (Table S15).

When stratified by age (<65 and ≥65), all blood cell counts remained significantly associated with mLOY at similar magnitude except for lymphocyte counts which were only marginally significant in the ≥65-year-old group (Tables S16–S19). As for NLR and TLR the magnitude of association between TLR and mLRR more than doubled in the ≥65 age group (Tables S20 and S21).

We also constructed a polygenic risk score using 156 SNPs previously found to be associated with mLOY^23^ as an instrumental variable to further investigate the association between mLOY and blood cell counts. As shown in Table S22, the mLOY PRS was significantly associated with leukocyte, erythrocyte, thrombocyte, monocyte, and neutrophil counts and the effect sizes closely mirrored those seen observationally for mLRR. Interestingly, the effect estimate for erythrocyte count was in the inverse direction for the mLOY PRS, suggesting potential non-genetic influences such as malnutrition or hypothyroidism could modify this association. The association between the mLOY PRS and blood cell counts was also examined in UK Biobank females and observed similar results as in males. We did not observe an association between mLOY PRS and overall lymphocyte count in the UK Biobank (Table S22).

In addition, we conducted reciprocal MR with SNPs associated with mLOY and blood cell counts (Table S23 and Fig. S4). We noted bi-directional effects for leukocyte count and thrombocyte count suggesting a potential shared biological process between mLOY and blood cell counts, although further studies are needed to confirm these findings. For erythrocyte counts, a potential effect was only observed from erythrocyte to mLOY, although the estimated effect size was small suggesting environmental or non-genetic contributors may also be important in this relationship.

Mediation analyses were also conducted to investigate whether mLOY acts as a potential mediator of the association between age or smoking and blood cell count. We found evidence suggesting most associations between age and blood cell counts were only partially mediated by mLOY (Tables S24 and S25). The estimated proportion of effect mediated by mLOY was less than 4%, suggesting any potential mediation effect of mLOY on age-related changes in blood cell counts only accounts for a small proportion of the total effect. We also found limited evidence suggesting mLOY mediated the association between smoking and blood cell counts (Tables S26 and S27) including leukocytes, erythrocytes, thrombocytes, monocytes and neutrophils with estimated proportion mediated of less than 2% of the total effect.

## Discussion

We present evidence from a large cross-sectional study that demonstrates significant population-based associations between men with mosaic loss of the Y chromosome (mLOY) and circulating blood counts, as measured in a complete blood count. While differences observed remain within the range of expected healthy counts, some men with high proportions of mLOY had substantial deviations in blood cell counts, which could be a harbinger of chronic disease risk.

In a 2018 report by Loh *et al*., enrichment of autosomal mosaic events was observed among people with abnormal blood cell indices in UK Biobank^63^. For instance, the odds of having a copy number neutral mosaic event in chromosome 9p was 17.7 [10.2, 30.6] times higher among people with top 1% erythrocyte counts (*p* = 1.1 × 10^−13^). While Loh *et al*. detected an interesting association, the authors only examined the extreme 1% of blood cell indices which resulted in a small sample size and accordingly wide confidence intervals. Their analysis was also a univariable statistical test which did not take into account potential confounding by other factors. A recently published study on 57, 987 men in Biobank Japan also reported associations among mLOY, increased thrombocyte and leukocyte counts^64^. While the authors did not find statistically significant negative associations between erythrocyte counts and continuous mLRR in univariable models, effect estimates indicate men with mLOY trended toward a decreased erythrocyte count. In the current study, we modeled mLOY in both continuous and categorical measures and employed multiple strategies to prevent potential confounding including multivariable models adjusting for potential risk factors as well as polygenic risk scores and a Mendelian randomization framework which are less susceptible to confounding.

A proposed biologic mechanism relating mLOY to disease risk has been through alteration of the immune system and its response to multiple factors^3,65^. mLOY could be associated with differences in blood counts either as a consequence of the mosaicism or as a response to one or more exposures that drive development and maintenance of mLOY. In particular, neutrophils have been suggested to promote tumor initiation by releasing reactive oxygen species, reactive nitrogen species, and proteases, as well as promote progression by activating senescent cancer cells and suppressing CD8+ T cell-mediated immune responses^66^. The role of thrombocytes is complex: classically platelet counts can be elevated in inflammatory conditions or select pediatric cancers (e.g., neuroblastoma). The literature also supports the association of elevated platelet counts with lung cancer risk^59^. Still it is plausible that thrombocytes could contribute to tumor progression by stimulating angiogenesis^67^ and activating thrombosis-related inflammation^68^. In addition, observational studies have found that the composition of blood cells including neutrophil-lymphocyte ratio and thrombocyte-lymphocyte ratio are associated with overall and disease-free survival in multiple cancers^69^.

Stratified analyses and inclusion of interaction terms in multivariable models demonstrated that smoking status interacts with mLOY and strengthened the association between mLOY and blood cell counts. Among the six blood cell types we reported, smoking status did not alter the directionality of associations between blood cell counts and mLOY except for lymphocytes. While mLOY was positively associated with lymphocyte counts in ever smokers, the association flipped in never smokers. This stark change in association along with a relatively mild association between mLOY and thrombocyte resulted in a larger effect size in association between TLR and mLOY in never smokers. Although high TLR has been suggested to be a prognostic biomarker reflecting systemic inflammation in cancer patients, the clinical utility of such a biomarker in healthy individuals remained uncertain. If deviations in blood cell counts were persistent over years or decades, these alterations could have an impact on immune regulation and immunosenscence, both likely contributors to chronic disease risk. Since we do not fully understand the underlying mechanisms, it is plausible that perturbations in genomic stability and cell cycle pathways could be altered as well. The strongest effect of mLOY is seen in the myeloid lineage, which is consistent with the immune hypothesis as a key element. The involvement of the myeloid lineage also suggests CHIP (a correlated phenotype) may have some relevance for these observed associations. We observed no relationship with basophil and eosinophil counts as well as overall lymphocyte counts.

It is notable that mLOY is associated with other non-leukocyte parameters, such as an increase in erythrocyte size and a decrease in hemoglobin concentration per cell as well as an increase in thrombocyte counts accompanies by a decrease in platelet distribution width. The changes in erythrocyte indices were similar to macrocytic hypochromic anemia. And the most common causes for macrocytic anemia were vitamin B12 and folate deficiency^70^. A report in 2016 on National Diet and Nutrition Surveys found that 12.4% and 6.4% women in childbearing age were deficient in serum vitamin B12 and folate despite 96% consumption of adequate B12 in UK^71^ suggesting that the current recommended intake of vitamin B12 and folate might require adjustment to accommodate difference in life style or genetic background which may influence bioavailability. The intake and deficiency of vitamin B12 and folate among men was under-studied. The cross-sectional design of current investigation is inadequate for elucidating causal relationship, but the potential involvement of mLOY and vitamin B12 and folate metabolism might warrant further study. The effect of mLOY on red blood cells is intriguing in light of the steady decline in hematopoietic regeneration after middle age- and indeed, our finding could be correlated but not causally related to such^72^.

Despite careful consideration of the effects of age and tobacco use in our analysis, we cannot rule out potential biases of residual confounding, or confounding by other unmeasured or unadjusted exposures. Increasing age and tobacco use are associated with both mLOY and changes in blood cell count indices and warrant careful consideration to remove potential confounding in our analysis of the association between mLOY and blood cell counts. To adjust for these effects, multivariable regression models adjusted for a 25-level smoking variable and allowed for non-linear relationships with age. Furthermore, stratified analyses restricted to strata of age group and smoking status found no major differences in overall analytical outcome except for in sub-analyses of lymphocytes; suggesting potential effect modification by age and tobacco use for lymphocytes. Additionally, we used a mLOY PRS as a genetic instrumental variable to further investigate the association between mLOY and blood cell counts. Again, blood cell indices that were significantly associated with mLOY in the observational data were also associated in this genetic analysis. The only exception was erythrocyte indices which remained highly significant but had effect sizes in the opposite direction, suggesting environmental contributors may have strong influence in this relationship.

Our analysis in the UK Biobank provides important evidence that mLOY in circulating blood cells is associated with changes in blood cell counts. Our cross-sectional investigation is unable to determine the temporality of this relationship (e.g., does mLOY precede changes in blood cell count). Results from our mediation analyses indicate mLOY could account for a small proportion of age and smoking-related effects on blood cell count; suggesting mLOY may precede changes in blood cell counts. Alternatively, the PRS analysis in women (who do not carry a Y chromosome) demonstrates a strong relationship between mLOY genetic susceptibility variants and blood cell counts independent of chromosome Y loss, suggesting blood cell count and mLOY may share common genetic risk factors related to genomic instability and cell cycle regulation or alternatively that genetic susceptibility to mLOY in men may be associated with genetic susceptibility to chromosomal alterations in women (e.g., mosaic chromosome X loss) that may have similar impacts on blood cell counts. Regardless, our analysis suggests mLOY and blood cell counts are highly associated and are relevant for underlying disease risk. Although the specific mechanisms responsible for the association between mLOY and blood cell counts are unclear, our work highlights the need to explore the functional bases of the reported associations. Future studies that examine the molecular impact of mLOY on cellular transcription, cell cycle regulation and differentiation are vital for expanding our understanding of how mLOY could have an impact on hematopoiesis, particularly in the aging population, and could provide novel insights into potential biological mechanisms responsible for the observed associations between mLOY and possible cancer and chronic disease risk.

### Transparency statement

The lead author (the manuscript’s guarantor) affirms that this manuscript is an honest, accurate, and transparent account of the study being reported; that no important aspects of the study have been omitted; and that any discrepancies from the study as originally planned (and, if relevant, registered) have been explained.

### Patient and public involvement

The development of the research question or outcome measures was not informed by patients’ priorities, experience, or preferences. No patients were involved in the design and conduct of the present study. There are no plans to disseminate the results to study participants.

## Supplementary information

Supplementary Information.

## Data Availability

The data reported in this paper are available by application directly to the UK Biobank. The associations between mLOY and outcomes are provided in the supplementary data. Software code in R for the analyses is available upon request.
