# Supplementary material for: Mosaic chromosome Y loss is associated with alterations in blood cell counts in UK Biobank men
Source: Sci Rep. 2020 Feb 27;10:3655. doi: 10.1038/s41598-020-59963-8 (PMC7046668; doi:10.1038/s41598-020-59963-8)
Supplement: Supplementary file 1 — Supplementary Information. [file 41598_2020_59963_MOESM1_ESM.docx]

**Supplementary materials**

**Table S1. Prevalence of mLOY by assessment centre and region**

| **Region/centre** | **# male participants** | **# males with mLOY males^a^** | **mLOY prevanlence (%)** |
| --- | --- | --- | --- |
| **England** | 183291 | 35524 | 19.38 |
| Birmingham | 10642 | 2140 | 20.11 |
| Bristol | 17329 | 3251 | 18.76 |
| Bury | 12305 | 2485 | 20.2 |
| Croydon | 10625 | 1950 | 18.35 |
| Hounslow | 11500 | 2061 | 17.92 |
| Leeds | 18288 | 3493 | 19.1 |
| Liverpool | 13617 | 2748 | 20.18 |
| London | 5125 | 791 | 15.43 |
| Manchester | 6095 | 1056 | 17.33 |
| Middlesbrough | 8914 | 1847 | 20.72 |
| Newcastle upon Tyne | 15332 | 2919 | 19.04 |
| Nottingham | 14031 | 2873 | 20.48 |
| Oxford | 5467 | 1037 | 18.97 |
| Reading | 12144 | 2319 | 19.1 |
| Sheffield | 12625 | 2704 | 21.42 |
| Stockport | 200 | 62 | 31 |
| Stoke-on-Trent | 9052 | 1788 | 19.75 |
| **Scotland** | 14492 | 2643 | 18.24 |
| Edinburgh | 6790 | 1226 | 18.06 |
| Glasgow | 7702 | 1417 | 18.4 |
| **Wales** | 8574 | 1642 | 19.15 |
| Cardiff | 7432 | 1396 | 18.78 |
| Swansea | 887 | 201 | 22.66 |
| Wrexham | 255 | 45 | 17.65 |

^a^Dichotomized mLOY

**Table S2. Association between mLOY and immune-related diseases**

| ICD10 code | Description | Odds Ratio (95% CI) | *P^a^* |
| --- | --- | --- | --- |
| D80.1 | Nonfamilial hypogammaglobulinaemia | 0.407 [0.06-1.387] | 0.171 |
| D86.0 | Sarcoidosis of lung | 0.892 [0.519-1.449] | 0.658 |
| D86.8 | Sarcoidosis of other and combined sites | 1.535 [0.757-2.899] | 0.223 |
| D86.9 | Sarcoidosis, unspecified | 0.993 [0.676-1.418] | 0.97 |
| D89.2 | Hypergammaglobulinaemia, unspecified | 0.842 [0.499-1.347] | 0.488 |

^a^Logistic regression with disease as outcome and binary mLOY status as predictor was performed. The model contained only mLOY and intercept.

**Table S3. Associations between leukocyte, erythrocyte, and thrombocyte count and mLOY**

|  | Leukocyte Count (×10^9^ cells/L)^a^ | | Erythrocyte Count (×10^12^ cells/L)^a^ | | Thrombocyte Count (×10^9^ cells/L)^a^ | |
| --- | --- | --- | --- | --- | --- | --- |
|  | Estimate (95% CI) | *P* | Estimate (95% CI) | *P* | Estimate (95% CI) | *P* |
| **mLOY**^b^ |  |  |  |  |  |  |
| Categorical | 0.218 [0.198, 0.239] | 9.22×10^-95^ | -0.009 [-0.014, -0.005] | 2.75×10^-5^ | 5.523 [4.862, 6.183] | 2.32×10^-60^ |
| Continuous^c^ | 0.058 [0.05, 0.066] | 6.48×10^-45^ | -0.009 [-0.01, -0.007] | 8.73×10^-23^ | 2.321 [2.063, 2.579] | 2.41×10^-69^ |
| **Age**^d^ |  |  |  |  |  |  |
| ≥65 years | 0.198 [0.178, 0.218] | 2.69×10^-80^ | -0.058 [-0.063, -0.054] | 1.98×10^-155^ | -9.905 [-10.557, -9.252] | 3.72×10^-194^ |
| Continuous | 0.014 [0.013, 0.015] | 1.69×10^-145^ | -0.005 [-0.005, -0.005] | < 5×10^-324^ | -0.681 [-0.715, -0.647] | < 5×10^-324^ |
| **Race/Ethnicity** |  |  |  |  |  |  |
| Mixed | -0.011 [-0.12, 0.098] | 0.841 | 0.075 [0.052, 0.098] | 1.55×10^-10^ | -1.56 [-5.029, 1.91] | 0.378 |
| Asian | 0.303 [0.252, 0.353] | 4.95×10^-32^ | 0.189 [0.179, 0.2] | 4.72×10^-267^ | 0.412 [-1.194, 2.018] | 0.615 |
| Black | -1.285 [-1.35, -1.221] | < 5×10^-324^ | 0.105 [0.091, 0.118] | 7.58×10^-52^ | -24.81 [-26.859, -22.761] | 2.47×10^-124^ |
| Other | -0.223 [-0.306, -0.139] | 1.71×10^-7^ | 0.16 [0.143, 0.178] | 1.23×10^-71^ | -7.842 [-10.502, -5.182] | 7.58×10^-9^ |
| **Smoking status**^e^ |  |  |  |  |  |  |
| Current smoker | 1.478 [1.453, 1.502] | < 5×10^-324^ | -0.057 [-0.062, -0.052] | 5.34×10^-103^ | 8.836 [8.056, 9.616] | 5.47×10^-109^ |
| Former smoker | 0.143 [0.126, 0.161] | 2.88×10^-59^ | -0.031 [-0.034, -0.027] | 2.24×10^-62^ | 3.965 [3.42, 4.511] | 5.23×10^-46^ |
| **Alcohol drinking** |  |  |  |  |  |  |
| Former drinker | 0.033 [-0.03, 0.097] | 0.298 | -0.068 [-0.082, -0.055] | 5.44×10^-24^ | -0.961 [-2.973, 1.051] | 0.349 |
| Occasional | 0.034 [-0.021, 0.089] | 0.229 | -0.012 [-0.024, -5.64×10^-4^] | 0.04 | -1.976 [-3.735, -0.217] | 0.028 |
| 1-3 drink/month | -0.016 [-0.07, 0.039] | 0.57 | -0.013 [-0.024, -0.001] | 0.03 | -1.679 [-3.413, 0.055] | 0.058 |
| 1-2 drink/week | -0.092 [-0.143, -0.042] | 3.41×10^-4^ | -0.051 [-0.061, -0.04] | 7.80×10^-21^ | -1.914 [-3.522, -0.305] | 0.02 |
| 3-4 drink/week | -0.225 [-0.276, -0.175] | 2.91×10^-18^ | -0.087 [-0.097, -0.076] | 4.14×10^-57^ | -2.993 [-4.608, -1.379] | 2.80×10^-4^ |
| Daily | -0.276 [-0.327, -0.225] | 2.27×10^-26^ | -0.122 [-0.132, -0.111] | 1.65×10^-109^ | -3.31 [-4.934, -1.686] | 6.48×10^-5^ |
| **Body mass index** |  |  |  |  |  |  |
| < 18.5 | 0.268 [0.101, 0.434] | 0.002 | -0.221 [-0.256, -0.186] | 5.70×10^-35^ | 6.818 [1.511, 12.125] | 0.012 |
| 25 to <30 | 0.241 [0.222, 0.26] | 1.62×10^-135^ | 0.096 [0.092, 0.1] | < 5×10^-324^ | -1.348 [-1.956, -0.74] | 1.40×10^-5^ |
| 30 to <35 | 0.512 [0.489, 0.536] | < 5×10^-324^ | 0.142 [0.137, 0.147] | < 5×10^-324^ | -3.881 [-4.641, -3.122] | 1.23×10^-23^ |
| >35 | 0.926 [0.889, 0.963] | < 5×10^-324^ | 0.139 [0.131, 0.147] | 7.50×10^-270^ | -4.554 [-5.727, -3.38] | 2.80×10^-14^ |
| Continuous | 0.06 [0.058, 0.062] | < 5×10^-324^ | 0.012 [0.012, 0.012] | < 5×10^-324^ | -0.325 [-0.386, -0.264] | 2.62×10^-25^ |
| **Diabetes** | 0.258 [0.224, 0.292] | 8.64×10^-51^ | -0.106 [-0.113, -0.098] | 2.63×10^-186^ | -3.049 [-4.124, -1.973] | 2.76×10^-8^ |
| **Hypertension** | 0.233 [0.214, 0.251] | 3.30×10^-137^ | 0.003 [-4.7×10^-4^, 0.007] | 0.085 | 3.112 [2.53, 3.694] | 1.14×10^-25^ |
| **Hypercholesterolemia** | 0.141 [0.118, 0.163] | 3.43×10^-35^ | -0.019 [-0.023, -0.014] | 6.17×10^-15^ | -2.343 [-3.054, -1.633] | 1.02×10^-10^ |

^a^Mean and standard deviation in subjects without mLOY: leukocyte (6.81, 1.85); erythrocyte (4.76, 0.38); thrombocyte (237.82, 55.54)

^b^P-values from multivariable linear regression models adjusted for all other variables in this table. Unless specified otherwise, we adjusted for age, age squared, race/ethnicity, smoking, alcohol consumption, body mass index (continuous), diabetes, hypertension, and hypercholesterolemia. Except for mLRR row, the reference group for categorical variables were no mLOY, white, never smoker, never drinker, 18.5 ≤ body mass index < 25, no diabetes, no hypertension, no hypercholesteroemia.
^c^The continuous variable reports the coefficient when mLRR was standardized.
^d^The continuous variable reports the coefficient for age without the squared term.
^e^Smoking status was adjusted here for never, former, and current smoker. The model for other rows adjusted smoking status by a 25-level detailed classification.

**Table S4. Associations between lymphocyte, monocyte, and neutrophil count and mLOY**

|  | Lymphocyte Count (×10^9^ cells/L)^a^ | | Monocyte Count (×10^9^ cells/L) | | Neutrophil Count (×10^9^ cells/L) | |
| --- | --- | --- | --- | --- | --- | --- |
|  | Estimate (95% CI) | *P* | Estimate (95% CI) | *P* | Estimate (95% CI) | *P* |
| **mLOY**^b^ |  |  |  |  |  |  |
| Categorical | 0.016 [0.007, 0.025] | 8.52×10^-4^ | 0.021 [0.018, 0.024] | 6.93×10^-57^ | 0.174 [0.158, 0.19] | 1.24×10^-99^ |
| Continuous^c^ | -0.002 [-0.005, 0.002] | 0.345 | 0.005 [0.004, 0.006] | 5.24×10^-25^ | 0.055 [0.048, 0.061] | 4.81×10^-65^ |
| **Age**^d^ |  |  |  |  |  |  |
| ≥65 years | -0.017 [-0.027, -0.008] | 1.92×10^-4^ | 0.028 [0.025, 0.03] | 7.47×10^-101^ | 0.183 [0.167, 0.199] | 6.44×10^-113^ |
| Continuous | -8.31×10^-4^ [-0.001, -3.52×10^-4^] | 6.78×10^-4^ | 0.002 [0.002, 0.002] | 3.92×10^-230^ | 0.012 [0.012, 0.013] | 1.58×10^-188^ |
| **Race/Ethnicity** |  |  |  |  |  |  |
| Mixed | 0.117 [0.068, 0.166] | 2.67×10^-6^ | -0.019 [-0.033, -0.006] | 0.006 | -0.121 [-0.205, -0.036] | 0.005 |
| Asian | 0.332 [0.309, 0.354] | 3.76×10^-181^ | -0.005 [-0.011, 0.001] | 0.124 | -0.084 [-0.123, -0.044] | 2.96×10^-5^ |
| Black | 0.154 [0.125, 0.183] | 1.42×10^-25^ | -0.101 [-0.109, -0.093] | 4.25×10^-132^ | -1.323 [-1.373, -1.273] | < 5×10^-324^ |
| Other | 0.188 [0.151, 0.226] | 5.78×10^-23^ | -0.045 [-0.055, -0.034] | 4.61×10^-17^ | -0.369 [-0.434, -0.304] | 7.17×10^-29^ |
| **Smoking status**^e^ |  |  |  |  |  |  |
| Current smoker | 0.361 [0.35, 0.372] | < 5×10^-324^ | 0.068 [0.064, 0.071] | < 5×10^-324^ | 1.002 [0.982, 1.021] | < 5×10^-324^ |
| Former smoker | 0.047 [0.04, 0.055] | 2.06×10^-33^ | 0.011 [0.008, 0.013] | 7.31×10^-22^ | 0.078 [0.064, 0.091] | 1.11×10^-29^ |
| **Alcohol drinking** |  |  |  |  |  |  |
| Former drinker | 0.004 [-0.024, 0.033] | 0.756 | -0.009 [-0.017, -9.42×10^-4^] | 0.028 | 0.031 [-0.018, 0.08] | 0.214 |
| Occasional | 0.019 [-0.005, 0.044] | 0.126 | -0.004 [-0.011, 0.003] | 0.258 | 0.026 [-0.017, 0.069] | 0.24 |
| 1-3 drink/month | 0.017 [-0.008, 0.041] | 0.176 | -7.09×10^-4^ [-0.008, 0.006] | 0.838 | -0.028 [-0.07, 0.015] | 0.202 |
| 1-2 drink/week | 0.011 [-0.011, 0.034] | 0.328 | -0.007 [-0.013, -7.79×10^-4^] | 0.028 | -0.089 [-0.128, -0.049] | 9.42×10^-6^ |
| 3-4 drink/week | -0.007 [-0.03, 0.016] | 0.544 | -0.011 [-0.017, -0.004] | 9.50×10^-4^ | -0.197 [-0.237, -0.158] | 1.04×10^-22^ |
| Daily | -0.034 [-0.057, -0.011] | 0.004 | -0.004 [-0.011, 0.002] | 0.174 | -0.226 [-0.266, -0.187] | 4.59×10^-29^ |
| **Body mass index** |  |  |  |  |  |  |
| < 18.5 | -0.208 [-0.282, -0.134] | 4.43×10^-8^ | 0.009 [-0.011, 0.03] | 0.376 | 0.301 [0.171, 0.431] | 5.47×10^-6^ |
| 25 to <30 | 0.123 [0.114, 0.131] | 4.74×10^-175^ | 0.023 [0.021, 0.026] | 1.79×10^-81^ | 0.086 [0.071, 0.101] | 4.72×10^-30^ |
| 30 to <35 | 0.241 [0.231, 0.252] | < 5×10^-324^ | 0.054 [0.051, 0.057] | 6.52×10^-277^ | 0.197 [0.179, 0.216] | 1.84×10^-96^ |
| >35 | 0.314 [0.298, 0.331] | 4.40×10^-307^ | 0.086 [0.082, 0.091] | 9.92×10^-295^ | 0.491 [0.463, 0.52] | 1.60×10^-247^ |
| Continuous | 0.023 [0.023, 0.024] | < 5×10^-324^ | 0.006 [0.006, 0.006] | < 5×10^-324^ | 0.029 [0.027, 0.03] | 1.98×10^-312^ |
| **Diabetes** | 0.038 [0.022, 0.053] | 1.14×10^-6^ | 0.005 [6.13×10^-4^, 0.009] | 0.025 | 0.199 [0.173, 0.225] | 7.52×10^-50^ |
| **Hypertension** | -0.012 [-0.021, -0.004] | 0.003 | 0.02 [0.018, 0.023] | 1.55×10^-67^ | 0.221 [0.207, 0.235] | 1.12×10^-203^ |
| **Hypercholesterolemia** | 0.043 [0.033, 0.053] | 8.15×10^-17^ | 0.012 [0.009, 0.015] | 6.26×10^-17^ | 0.083 [0.066, 0.101] | 5.08×10^-21^ |

^a^Mean and standard deviation in subjects without mLOY: lymphocyte (1.89, 0.81); monocyte (0.51, 0.22); neutrophil (4.19, 1.41)

^b^P-values from multivariable linear regression models adjusted for all other variables in this table. Unless specified otherwise, we adjusted for age, age squared, race/ethnicity, smoking, alcohol consumption, body mass index (continuous), diabetes, hypertension, and hypercholesterolemia. Except for mLRR row, the reference group for categorical variables were no mLOY, white, never smoker, never drinker, 18.5 ≤ body mass index < 25, no diabetes, no hypertension, no hypercholesteroemia.
^c^The continuous variable reports the coefficient when mLRR was standardized.
^d^The continuous variable reports the coefficient for age without the squared term.
^e^Smoking status was adjusted here for never, former, and current smoker. The model for other rows adjusted smoking status by a 25-level detailed classification.

**Table S5. Associations between leukocyte, erythrocyte, and thrombocyte count and mLOY with or without adjustment for immune-related diseases**

|  | Leukocyte Count (×109 cells/L)a | | Erythrocyte Count (×1012 cells/L)a | | Thrombocyte Count (×109 cells/L)a | |
| --- | --- | --- | --- | --- | --- | --- |
|  | Estimate (95% CI) | Pd | Estimate (95% CI) | P | Estimate (95% CI) | P |
| **Model without immune diseases^a^** | | | | | | |
| mLOY |  |  |  |  |  |  |
| Categorical | 0.218 [0.198, 0.239] | 9.22×10^-95^ | -0.009 [-0.014, -0.005] | 2.75×10^-5^ | 5.523 [4.862, 6.183] | 2.32×10^-60^ |
| Continuous^b^ | 0.058 [0.05, 0.066] | 6.48×10^-45^ | -0.009 [-0.01, -0.007] | 8.73×10^-23^ | 2.321 [2.063, 2.579] | 2.41×10^-69^ |
| **Model with immune diseases^c^** | | | | | | |
| **mLOY** |  |  |  |  |  |  |
| Categorical | 0.218 [0.198, 0.239] | 1.04×10^-94^ | -0.009 [-0.014, -0.005] | 2.75×10^-5^ | 5.524 [4.863, 6.184] | 2.21×10^-60^ |
| Continuous | 0.058 [0.05, 0.066] | 6.70×10^-45^ | -0.009 [-0.01, -0.007] | 8.69×10^-23^ | 2.321 [2.063, 2.579] | 2.42×10^-69^ |
| **D80.1^d^** | -0.023 [-0.714, 0.668] | 0.948 | 0.006 [-0.14, 0.151] | 0.939 | 8.954 [-13.074, 30.982] | 0.426 |
| **D86.0^d^** | -0.251 [-0.616, 0.114] | 0.178 | 0.023 [-0.054, 0.1] | 0.558 | 2.022 [-9.617, 13.661] | 0.733 |
| **D86.8^d^** | 0.371 [-0.166, 0.909] | 0.176 | 0.028 [-0.086, 0.141] | 0.634 | 2.141 [-14.993, 19.275] | 0.807 |
| **D86.9^d^** | 0.024 [-0.254, 0.302] | 0.865 | -0.018 [-0.077, 0.04] | 0.541 | 3.235 [-5.614, 12.084] | 0.474 |
| **D89.2^d^** | 0.008 [-0.312, 0.328] | 0.96 | 0.017 [-0.051, 0.084] | 0.627 | 5.044 [-5.156, 15.244] | 0.332 |

^a^P-values from multivariable linear regression models adjusted for all other variables in this table. Unless specified otherwise, we adjusted for age, age squared, race/ethnicity, smoking, alcohol consumption, body mass index (continuous), diabetes, hypertension, and hypercholesterolemia. Except for mLRR row, the reference group for categorical variables were no mLOY, white, never smoker, never drinker, 18.5 ≤ body mass index < 25, no diabetes, no hypertension, no hypercholesteroemia.
^b^The continuous variable reports the coefficient when mLRR was standardized
^c^This model adjusted for all five diseases listed in addition to the above model.
^d^D80.1 Nonfamilial hypogammaglobulinaemia. D86.0 Sarcoidosis of lung. D86.8 Sarcoidosis of other and combined sites. D86.9 Sarcoidosis, unspecified. D89.2 Hypergammaglobulinaemia, unspecified.

**Table S6. Associations between lymphocyte, monocyte, and neutrophil count and mLOY with or without adjustment for immune-related diseases**

|  | Lymphocyte Count (×10^9^ cells/L)^a^ | | Monocyte Count (×10^9^ cells/L) | | Neutrophil Count (×10^9^ cells/L) | |
| --- | --- | --- | --- | --- | --- | --- |
|  | Estimate (95% CI) | *P* | Estimate (95% CI) | Estimate (95% CI) | *P* | Estimate (95% CI) |
| **Model without immune diseases** | | | | | | |
| **mLOY**^a^ |  |  |  |  |  |  |
| Categorical | 0.016 [0.007, 0.025] | 8.52×10^-4^ | 0.021 [0.018, 0.024] | 6.93×10^-57^ | 0.174 [0.158, 0.19] | 1.24×10^-99^ |
| Continuous^b^ | -0.002 [-0.005, 0.002] | 0.345 | 0.005 [0.004, 0.006] | 5.24×10^-25^ | 0.055 [0.048, 0.061] | 4.81×10^-65^ |
| **Model with immune diseases^c^** | | | | | | |
| **mLOY** |  |  |  |  |  |  |
| Categorical | 0.016 [0.006, 0.025] | 8.74×10^-4^ | 0.021 [0.018, 0.024] | 6.70×10^-57^ | 0.174 [0.158, 0.19] | 1.33×10^-99^ |
| Continuous | -0.002 [-0.005, 0.002] | 0.345 | 0.005 [0.004, 0.006] | 5.12×10^-25^ | 0.055 [0.048, 0.061] | 5.05×10^-65^ |
| **D80.1^d^** | -0.108 [-0.418, 0.202] | 0.494 | 0.046 [-0.04, 0.133] | 0.294 | 0.053 [-0.483, 0.59] | 0.846 |
| **D86.0^d^** | -0.12 [-0.284, 0.044] | 0.151 | -0.036 [-0.081, 0.01] | 0.125 | -0.089 [-0.372, 0.195] | 0.54 |
| **D86.8^d^** | 0.174 [-0.066, 0.415] | 0.156 | -0.019 [-0.087, 0.048] | 0.573 | 0.185 [-0.233, 0.602] | 0.386 |
| **D86.9^d^** | -0.018 [-0.143, 0.106] | 0.773 | 0.016 [-0.019, 0.051] | 0.364 | 0.035 [-0.18, 0.251] | 0.748 |
| **D89.2^d^** | 0.049 [-0.094, 0.192] | 0.502 | -5.53×10^-4^ [-0.041, 0.039] | 0.978 | -0.047 [-0.295, 0.202] | 0.711 |

^a^P-values from multivariable linear regression models adjusted for all other variables in this table. Unless specified otherwise, we adjusted for age, age squared, race/ethnicity, smoking, alcohol consumption, body mass index (continuous), diabetes, hypertension, and hypercholesterolemia. Except for mLRR row, the reference group for categorical variables were no mLOY, white, never smoker, never drinker, 18.5 ≤ body mass index < 25, no diabetes, no hypertension, no hypercholesteroemia.
^b^The continuous variable reports the coefficient when mLRR was standardized
^c^This model adjusted for all five diseases listed in addition to the above model.
^d^D80.1 Nonfamilial hypogammaglobulinaemia. D86.0 Sarcoidosis of lung. D86.8 Sarcoidosis of other and combined sites. D86.9 Sarcoidosis, unspecified. D89.2 Hypergammaglobulinaemia, unspecified.

**Table S7. Associations between neutrophil-lymphocyte ratio (NLR), thrombocyte-lymphocyte ratio (TLR) and mLOY**

|  | NLR^a^ | | TLR | |
| --- | --- | --- | --- | --- |
|  | Estimate (95% CI) | *P* | Estimate (95% CI) | *P* |
| **mLOY** |  |  |  |  |
| Categorical | 0.061 [0.045, 0.077] | 2.98×10^-14^ | 1.288 [0.564, 2.012] | 4.90×10^-4^ |
| Continuous^b^ | 0.038 [0.032, 0.044] | 1.39×10^-33^ | 1.708 [1.424, 1.991] | 3.31×10^-32^ |
| **Age** |  |  |  |  |
| ≥65 years | 0.191 [0.175, 0.206] | 2.64×10^-128^ | -2.122 [-2.836, -1.408] | 5.75×10^-9^ |
| Continuous^c^ | 0.012 [0.011, 0.013] | 1.08×10^-178^ | -0.182 [-0.219, -0.145] | 1.40×10^-21^ |
| **Race/Ethnicity** |  |  |  |  |
| Mixed | -0.216 [-0.298, -0.133] | 3.10×10^-7^ | -8.79 [-12.595, -4.985] | 5.96×10^-6^ |
| Asian | -0.391 [-0.429, -0.353] | 3.46×10^-89^ | -19.539 [-21.302, -17.776] | 1.49×10^-104^ |
| Black | -0.88 [-0.929, -0.831] | 9.05×10^-273^ | -22.905 [-25.154, -20.657] | 1.30×10^-88^ |
| Other | -0.425 [-0.488, -0.361] | 1.53×10^-39^ | -16.886 [-19.8, -13.972] | 7.01×10^-30^ |
| **Smoking status**^d^ |  |  |  |  |
| Current smoker | 0.075 [0.056, 0.093] | 3.38×10^-15^ | -18.886 [-19.742, -18.03] | < 5×10^-324^ |
| Former smoker | -0.02 [-0.033, -0.007] | 0.003 | -1.37 [-1.969, -0.772] | 7.23×10^-6^ |
| **Alcohol drinking** |  |  |  |  |
| Former drinker | 0.029 [-0.019, 0.077] | 0.241 | -0.599 [-2.806, 1.608] | 0.595 |
| Occasional | -0.006 [-0.048, 0.036] | 0.78 | -1.922 [-3.852, 0.008] | 0.051 |
| 1-3 drink/month | -0.044 [-0.085, -0.002] | 0.038 | -2.27 [-4.173, -0.368] | 0.019 |
| 1-2 drink/week | -0.086 [-0.124, -0.048] | 1.06×10^-5^ | -2.749 [-4.513, -0.985] | 0.002 |
| 3-4 drink/week | -0.132 [-0.17, -0.093] | 1.98×10^-11^ | -2.484 [-4.255, -0.712] | 0.006 |
| Daily | -0.121 [-0.16, -0.082] | 8.81×10^-10^ | -0.997 [-2.779, 0.785] | 0.273 |
| **Body mass index** |  |  |  |  |
| < 18.5 | 0.682 [0.552, 0.812] | 1.04×10^-24^ | 27.164 [21.223, 33.105] | 3.22×10^-19^ |
| 25 to <30 | -0.148 [-0.163, -0.133] | 3.95×10^-84^ | -12.55 [-13.229, -11.871] | 2.29×10^-286^ |
| 30 to <35 | -0.233 [-0.252, -0.215] | 1.85×10^-133^ | -22.102 [-22.949, -21.254] | < 5×10^-324^ |
| >35 | -0.192 [-0.221, -0.163] | 3.23×10^-39^ | -28.233 [-29.543, -26.924] | < 5×10^-324^ |
| Continuous | -0.018 [-0.019, -0.016] | 1.61×10^-126^ | -2.096 [-2.163, -2.028] | < 5×10^-324^ |
| **Diabetes** | 0.102 [0.076, 0.127] | 7.02×10^-15^ | -1.85 [-3.03, -0.67] | 0.002 |
| **Hypertension** | 0.171 [0.157, 0.185] | 2.11×10^-129^ | 3.596 [2.958, 4.235] | 2.50×10^-28^ |
| **Hypercholesterolemia** | -0.013 [-0.03, 0.004] | 0.136 | -4.629 [-5.409, -3.85] | 2.59×10^-31^ |

^a^Mean and standard deviation in subjects without mLOY: NLR (2.422, 1.321); TLR (138.140, 62.578)

^b^P-values from multivariable linear regression models adjusted for all other variables in this table. Unless specified otherwise, we adjusted for age, age squared, race/ethnicity, smoking, alcohol consumption, body mass index (continuous variable), diabetes, hypertension, and hypercholesterolemia. Except for continuous variable rows, the reference group for categorical variables were no mLOY, White, never smoker, never drinker, 18.5 ≤ body mass index < 25, no diabetes, no hypertension, no hypercholesteroemia.
^b^The continuous variable reports the coefficient when mLRR was standardized.
^c^The continuous variable reports the coefficient for age without the squared term.
^d^Smoking status was adjusted here for never, former, and current smoker. The model for other rows adjusted smoking status by a 25-level detailed classification.

**Table S8. Associations between mean corpuscular volume (MCV), mean corpuscular hemoglobin (MCH), and mean corpuscular hemoglobin concentration (MCHC) and mLOY**

|  | MCV (fl)^a^ | | MCH (pg/cell) | | MCHC (g/dl) | |
| --- | --- | --- | --- | --- | --- | --- |
|  | Estimate (95% CI) | *P* | Estimate (95% CI) | *P* | Estimate (95% CI) | *P* |
| **mLOY** |  |  |  |  |  |  |
| Categorical | 0.231 [0.181, 0.281] | 1.94×10^-19^ | 0.059 [0.038, 0.079] | 4.36×10^-8^ | -0.022 [-0.035, -0.01] | 4.62×10^-4^ |
| Continuous^b^ | 0.127 [0.107, 0.147] | 1.02×10^-36^ | 0.033 [0.025, 0.041] | 4.50×10^-15^ | -0.012 [-0.017, -0.007] | 2.89×10^-6^ |
| **Age** |  |  |  |  |  |  |
| ≥65 years | 0.755 [0.705, 0.805] | 2.46×10^-194^ | 0.167 [0.147, 0.188] | 1.68×10^-56^ | -0.105 [-0.117, -0.092] | 1.71×10^-61^ |
| Continuous^c^ | 0.068 [0.065, 0.07] | < 5×10^-324^ | 0.017 [0.016, 0.018] | 6.85×10^-201^ | -0.007 [-0.008, -0.007] | 1.25×10^-112^ |
| **Race/Ethnicity** |  |  |  |  |  |  |
| Mixed | -1.42 [-1.684, -1.156] | 5.14×10^-26^ | -0.716 [-0.826, -0.606] | 3.13×10^-37^ | -0.261 [-0.327, -0.195] | 1.01×10^-14^ |
| Asian | -3.193 [-3.315, -3.07] | < 5×10^-324^ | -1.38 [-1.43, -1.329] | < 5×10^-324^ | -0.337 [-0.367, -0.306] | 4.15×10^-103^ |
| Black | -2.554 [-2.709, -2.398] | 7.26×10^-226^ | -1.592 [-1.657, -1.527] | < 5×10^-324^ | -0.819 [-0.858, -0.78] | < 5×10^-324^ |
| Other | -2.32 [-2.522, -2.118] | 8.38×10^-112^ | -1.138 [-1.223, -1.054] | 8.79×10^-154^ | -0.398 [-0.449, -0.348] | 1.45×10^-53^ |
| **Smoking status^d^** |  |  |  |  |  |  |
| Current smoker | 2.076 [2.016, 2.135] | < 5×10^-324^ | 0.692 [0.667, 0.717] | < 5×10^-324^ | -0.028 [-0.043, -0.013] | 2.12×10^-4^ |
| Former smoker | 0.157 [0.116, 0.199] | 1.19×10^-13^ | 0.048 [0.03, 0.065] | 7.02×10^-8^ | -0.007 [-0.018, 0.003] | 0.168 |
| **Alcohol drinking** |  |  |  |  |  |  |
| Former drinker | 0.437 [0.284, 0.59] | 2.10×10^-8^ | 0.152 [0.088, 0.216] | 3.13×10^-6^ | 0.003 [-0.035, 0.041] | 0.878 |
| Occasional | 0.267 [0.133, 0.401] | 9.17×10^-5^ | 0.088 [0.032, 0.143] | 0.002 | -0.003 [-0.036, 0.031] | 0.876 |
| 1-3 drink/month | 0.341 [0.209, 0.473] | 4.04×10^-7^ | 0.114 [0.059, 0.169] | 4.66×10^-5^ | -0.001 [-0.034, 0.032] | 0.94 |
| 1-2 drink/week | 0.85 [0.728, 0.972] | 3.01×10^-42^ | 0.35 [0.299, 0.401] | 3.99×10^-41^ | 0.065 [0.035, 0.096] | 2.92×10^-5^ |
| 3-4 drink/week | 1.5 [1.377, 1.623] | 1.44×10^-126^ | 0.616 [0.565, 0.668] | 7.87×10^-123^ | 0.11 [0.079, 0.14] | 2.78×10^-12^ |
| Daily | 2.36 [2.236, 2.483] | 7.42×10^-306^ | 0.944 [0.892, 0.995] | 2.72×10^-281^ | 0.142 [0.111, 0.173] | 2.37×10^-19^ |
| **Body mass index** |  |  |  |  |  |  |
| < 18.5 | 1.607 [1.204, 2.011] | 5.92×10^-15^ | 0.357 [0.189, 0.526] | 3.25×10^-5^ | -0.219 [-0.32, -0.118] | 2.10×10^-5^ |
| 25 to <30 | -0.559 [-0.606, -0.513] | 4.14×10^-124^ | -0.098 [-0.117, -0.078] | 3.29×10^-23^ | 0.104 [0.092, 0.115] | 5.03×10^-69^ |
| 30 to <35 | -0.798 [-0.856, -0.74] | 2.30×10^-161^ | -0.166 [-0.19, -0.142] | 1.49×10^-41^ | 0.121 [0.106, 0.135] | 3.69×10^-60^ |
| >35 | -0.903 [-0.992, -0.814] | 1.87×10^-87^ | -0.264 [-0.302, -0.227] | 5.45×10^-44^ | 0.051 [0.029, 0.074] | 6.63×10^-6^ |
| Continuous | -0.075 [-0.079, -0.07] | 2.23×10^-216^ | -0.02 [-0.022, -0.018] | 4.79×10^-90^ | 0.006 [0.005, 0.008] | 1.14×10^-26^ |
| **Diabetes** | -0.208 [-0.289, -0.126] | 6.41×10^-7^ | -0.135 [-0.169, -0.101] | 8.80×10^-15^ | -0.073 [-0.093, -0.052] | 3.04×10^-12^ |
| **Hypertension** | 0.056 [0.012, 0.101] | 0.013 | 0.047 [0.029, 0.066] | 5.45×10^-7^ | 0.027 [0.016, 0.039] | 1.17×10^-6^ |
| **Hypercholesterolemia** | -0.076 [-0.13, -0.022] | 0.006 | -0.048 [-0.071, -0.026] | 2.51×10^-5^ | -0.021 [-0.035, -0.008] | 0.002 |

^a^Mean and standard deviation in subjects without mLOY: MCV (91.188, 4.467); MCH (31.599, 1.847); MCHC (34.651, 1.054)

^b^P-values from multivariable linear regression models adjusted for all other variables in this table. Unless specified otherwise, we adjusted for age, age squared, race/ethnicity, smoking, alcohol consumption, body mass index (continuous variable), diabetes, hypertension, and hypercholesterolemia. Except for continuous variable rows, the reference group for categorical variables were no mLOY, White, never smoker, never drinker, 18.5 ≤ body mass index < 25, no diabetes, no hypertension, no hypercholesteroemia.
^b^The continuous variable reports the coefficient when mLRR was standardized.
^c^The continuous variable reports the coefficient for age without the squared term.
^d^Smoking status was adjusted here for never, former, and current smoker. The model for other rows adjusted smoking status by a 25-level detailed classification as described previously

**Table S9. Associations between mean sphered cell volume, (MSCV), immature reticulocyte fraction (IRF), and platelet distribution width (PDW) and mLOY**

|  | MSCV (fl)^a^ | | IRF (%) | | PDW (%) | |
| --- | --- | --- | --- | --- | --- | --- |
|  | Estimate (95% CI) | *P* | Estimate (95% CI) | *P* | Estimate (95% CI) | *P* |
| **mLOY** |  |  |  |  |  |  |
| Categorical | 0.315 [0.254, 0.376] | 5.37×10^-24^ | 0.217 [0.148, 0.286] | 6.33×10^-10^ | -0.025 [-0.031, -0.018] | 1.83×10^-14^ |
| Continuous | 0.123 [0.1, 0.147] | 4.31×10^-24^ | 0.149 [0.122, 0.176] | 1.75×10^-27^ | -0.004 [-0.007, -0.002] | 9.84×10^-4^ |
| **Age** |  |  |  |  |  |  |
| ≥65 years | 0.991 [0.931, 1.052] | 4.04×10^-226^ | 0.28 [0.212, 0.347] | 6.89×10^-16^ | 0.029 [0.023, 0.035] | 1.98×10^-20^ |
| Continuous | 0.085 [0.082, 0.089] | < 5×10^-324^ | 0.014 [0.011, 0.018] | 1.46×10^-15^ | 0.003 [0.002, 0.003] | 2.02×10^-62^ |
| **Race/Ethnicity** |  |  |  |  |  |  |
| Mixed | 0.718 [0.396, 1.039] | 1.21×10^-5^ | 1.08 [0.718, 1.443] | 5.25×10^-9^ | -0.04 [-0.073, -0.007] | 0.018 |
| Asian | -0.769 [-0.918, -0.62] | 4.65×10^-24^ | 1.325 [1.157, 1.493] | 7.97×10^-54^ | 0.003 [-0.012, 0.018] | 0.681 |
| Black | 3.27 [3.08, 3.46] | 1.26×10^-248^ | 3.745 [3.531, 3.96] | 4.17×10^-256^ | -0.097 [-0.116, -0.077] | 1.72×10^-22^ |
| Other | 0.103 [-0.144, 0.349] | 0.414 | 1.774 [1.496, 2.052] | 6.71×10^-36^ | 0.036 [0.011, 0.062] | 0.005 |
| **Smoking status** |  |  |  |  |  |  |
| Current smoker | 2.554 [2.482, 2.627] | < 5×10^-324^ | 0.98 [0.898, 1.061] | 4.08×10^-123^ | -0.069 [-0.077, -0.062] | 1.27×10^-75^ |
| Former smoker | 0.146 [0.096, 0.197] | 1.43×10^-8^ | 0.333 [0.276, 0.389] | 2.25×10^-30^ | 0.009 [0.004, 0.014] | 7.64×10^-4^ |
| **Alcohol drinking** |  |  |  |  |  |  |
| Former drinker | 0.497 [0.311, 0.683] | 1.58×10^-7^ | -0.251 [-0.461, -0.041] | 0.019 | -0.005 [-0.024, 0.014] | 0.595 |
| Occasional | 0.204 [0.041, 0.367] | 0.014 | -0.306 [-0.489, -0.122] | 0.001 | -0.01 [-0.027, 0.006] | 0.226 |
| 1-3 drink/month | 0.164 [0.004, 0.325] | 0.044 | -0.309 [-0.49, -0.128] | 8.02×10^-4^ | -0.013 [-0.03, 0.003] | 0.112 |
| 1-2 drink/week | 0.636 [0.487, 0.784] | 5.02×10^-17^ | -0.094 [-0.261, 0.074] | 0.273 | -0.016 [-0.031, -3.45×10^-4^] | 0.045 |
| 3-4 drink/week | 1.243 [1.094, 1.393] | 5.78×10^-60^ | 0.125 [-0.043, 0.293] | 0.145 | -0.022 [-0.037, -0.007] | 0.005 |
| Daily | 2.201 [2.051, 2.351] | 2.35×10^-181^ | 0.48 [0.311, 0.65] | 2.69×10^-8^ | -0.025 [-0.041, -0.01] | 0.001 |
| **Body mass index** |  |  |  |  |  |  |
| < 18.5 | 3.055 [2.564, 3.547] | 3.81×10^-34^ | -1.344 [-1.902, -0.787] | 2.28×10^-6^ | -0.005 [-0.056, 0.045] | 0.839 |
| 25 to <30 | -1.086 [-1.142, -1.03] | 1.37×10^-313^ | 1.827 [1.763, 1.891] | < 5×10^-324^ | 0.042 [0.036, 0.047] | 4.84×10^-45^ |
| 30 to <35 | -1.477 [-1.547, -1.407] | < 5×10^-324^ | 3.636 [3.556, 3.715] | < 5×10^-324^ | 0.071 [0.064, 0.078] | 1.19×10^-82^ |
| >35 | -1.434 [-1.542, -1.325] | 2.38×10^-148^ | 5.531 [5.409, 5.654] | < 5×10^-324^ | 0.082 [0.071, 0.093] | 1.42×10^-46^ |
| Continuous | -0.127 [-0.133, -0.122] | < 5×10^-324^ | 0.386 [0.379, 0.392] | < 5×10^-324^ | 0.006 [0.005, 0.007] | 3.06×10^-92^ |
| **Diabetes** | -0.25 [-0.35, -0.151] | 8.10×10^-7^ | 1.03 [0.918, 1.142] | 2.51×10^-72^ | 0.058 [0.048, 0.068] | 1.18×10^-28^ |
| **Hypertension** | 0.048 [-0.006, 0.102] | 0.081 | 0.476 [0.415, 0.537] | 2.62×10^-53^ | 0.004 [-0.001, 0.01] | 0.125 |
| **Hypercholesterolemia** | -0.623 [-0.688, -0.557] | 4.75×10^-77^ | 1.053 [0.978, 1.127] | 2.28×10^-170^ | 4.3×10^-4^ [-0.006, 0.007] | 0.9 |

^a^Mean and standard deviation in subjects without mLOY: MSCV (82.489, 5.247); IRF (28.985, 6.044); PDW (16.568, 0.530);

^b^P-values from multivariable linear regression models adjusted for all other variables in this table. Unless specified otherwise, we adjusted for age, age squared, race/ethnicity, smoking, alcohol consumption, body mass index (continuous variable), diabetes, hypertension, and hypercholesterolemia. Except for continuous variable rows, the reference group for categorical variables were no mLOY, White, never smoker, never drinker, 18.5 ≤ body mass index < 25, no diabetes, no hypertension, no hypercholesteroemia.
^b^The continuous variable reports the coefficient when mLRR was standardized.
^c^The continuous variable reports the coefficient for age without the squared term.
^d^Smoking status was adjusted here for never, former, and current smoker. The model for other rows adjusted smoking status by a 25-level detailed classification as described previously

**Table S10. Associations between leukocyte, erythrocyte, and platelet counts by mLOY status in ever smokers**

|  | Leukocyte Count (×10^9^ cells/L) | | Erythrocyte Count (×10^12^ cells/L) | | Thrombocyte Count (×10^9^ cells/L) | |
| --- | --- | --- | --- | --- | --- | --- |
|  | Estimate (95% CI) | *P*^a^ | Estimate (95% CI) | *P* | Estimate (95% CI) | *P* |
| **mLOY** |  |  |  |  |  |  |
| Categorical | 0.486 [0.456, 0.515] | 2.45×10^-228^ | -0.019 [-0.025, -0.013] | 7.34×10^-11^ | 7.054 [6.179, 7.929] | 3.34×10^-56^ |
| Continuous^b^ | 0.136 [0.125, 0.147] | 5.99×10^-126^ | -0.013 [-0.015, -0.011] | 2.79×10^-32^ | 2.651 [2.321, 2.982] | 1.23×10^-55^ |
| **Age**^c^ |  |  |  |  |  |  |
| ≥65 years | -0.032 [-0.062, -0.003] | 0.029 | -0.048 [-0.054, -0.043] | 2.58×10^-62^ | -10.483 [-11.349, -9.617] | 4.08×10^-124^ |
| Continuous | -0.006 [-0.008, -0.004] | 4.86×10^-13^ | -0.004 [-0.005, -0.004] | 1.98×10^-153^ | -0.748 [-0.796, -0.7] | 1.16×10^-202^ |
| **Race/Ethnicity** |  |  |  |  |  |  |
| Mixed | 0.073 [-0.089, 0.235] | 0.376 | 0.077 [0.045, 0.108] | 1.90×10^-6^ | -0.005 [-4.809, 4.798] | 0.998 |
| Asian | 0.136 [0.044, 0.229] | 0.004 | 0.201 [0.184, 0.219] | 5.85×10^-107^ | -4.465 [-7.204, -1.725] | 0.001 |
| Black | -1.21 [-1.322, -1.099] | 1.81×10^-100^ | 0.068 [0.046, 0.09] | 7.28×10^-10^ | -18.681 [-21.991, -15.371] | 1.98×10^-28^ |
| Other | -0.224 [-0.353, -0.095] | 6.55×10^-4^ | 0.136 [0.111, 0.161] | 1.72×10^-26^ | -8.266 [-12.094, -4.438] | 2.32×10^-5^ |
| **Alcohol drinking** |  |  |  |  |  |  |
| Former drinker | -0.1 [-0.224, 0.024] | 0.114 | -0.088 [-0.112, -0.064] | 8.49×10^-13^ | -1.983 [-5.662, 1.695] | 0.291 |
| Occasional | 0.04 [-0.078, 0.158] | 0.507 | -0.025 [-0.048, -0.002] | 0.032 | -1.504 [-5.023, 2.016] | 0.402 |
| 1-3 drink/month | -0.152 [-0.27, -0.035] | 0.011 | -0.024 [-0.047, -0.001] | 0.039 | -3.457 [-6.959, 0.045] | 0.053 |
| 1-2 drink/week | -0.364 [-0.476, -0.251] | 2.62×10^-10^ | -0.065 [-0.087, -0.043] | 6.47×10^-9^ | -3.936 [-7.287, -0.585] | 0.021 |
| 3-4 drink/week | -0.618 [-0.73, -0.505] | 6.70×10^-27^ | -0.101 [-0.123, -0.079] | 1.60×10^-19^ | -5.969 [-9.319, -2.62] | 4.78×10^-4^ |
| Daily | -0.567 [-0.679, -0.455] | 4.66×10^-23^ | -0.143 [-0.165, -0.121] | 1.16×10^-37^ | -5.84 [-9.18, -2.501] | 6.09×10^-4^ |
| **Body mass index** |  |  |  |  |  |  |
| < 18.5 | 0.876 [0.639, 1.114] | 5.08×10^-13^ | -0.217 [-0.263, -0.171] | 5.07×10^-20^ | 17.385 [10.3, 24.47] | 1.51×10^-6^ |
| 25 to <30 | 0.044 [0.013, 0.074] | 0.005 | 0.095 [0.089, 0.101] | 1.01×10^-213^ | -4.123 [-5.034, -3.211] | 7.74×10^-19^ |
| 30 to <35 | 0.264 [0.228, 0.301] | 1.01×10^-45^ | 0.143 [0.136, 0.15] | < 5×10^-324^ | -7.673 [-8.76, -6.585] | 1.95×10^-43^ |
| >35 | 0.621 [0.566, 0.675] | 6.66×10^-110^ | 0.139 [0.128, 0.149] | 1.58×10^-143^ | -9.668 [-11.293, -8.043] | 2.19×10^-31^ |
| Continuous | 0.036 [0.033, 0.039] | 2.76×10^-131^ | 0.012 [0.011, 0.012] | < 5×10^-324^ | -0.708 [-0.795, -0.622] | 4.98×10^-58^ |
| **Diabetes** | 0.267 [0.219, 0.314] | 6.38×10^-28^ | -0.103 [-0.112, -0.093] | 9.48×10^-104^ | -3.096 [-4.513, -1.679] | 1.85×10^-5^ |
| **Hypertension** | 0.233 [0.206, 0.261] | 3.54×10^-63^ | -0.005 [-0.01, 2.49×10^-4^] | 0.062 | 3.491 [2.682, 4.301] | 2.87×10^-17^ |
| **Hypercholesterolemia** | 0.189 [0.157, 0.221] | 1.00×10^-30^ | -0.023 [-0.03, -0.017] | 2.84×10^-13^ | -1.706 [-2.663, -0.75] | 4.70×10^-4^ |

^a^P-values from multivariable linear regression models adjusted for all other variables in this table. Unless specified otherwise, we adjusted for age, age squared, race/ethnicity, alcohol consumption, body mass index (continuous variable), diabetes, hypertension, and hypercholesterolemia. Except for continuous variable rows, the reference group for categorical variables were no mLOY, White, never drinker, 18.5 ≤ body mass index < 25, no diabetes, no hypertension, no hypercholesteroemia.
^b^The continuous variable reports the coefficient when mLRR was standardized.
^c^The continuous variable reports the coefficient for age without the squared term.

**Table S11. Linear regression for lymphocyte, monocyte and neutrophil counts by mLOY status in ever smokers**

|  | Lymphocyte Count (×10^9^ cells/L) | | Monocyte Count (×10^9^ cells/L) | | Neutrophil Count (×10^9^ cells/L) | |
| --- | --- | --- | --- | --- | --- | --- |
|  | Estimate (95% CI) | *P*^a^ | Estimate (95% CI) | *P* | Estimate (95% CI) | *P* |
| **mLOY** |  |  |  |  |  |  |
| Categorical | 0.082 [0.07, 0.094] | 1.36×10^-40^ | 0.033 [0.029, 0.037] | 1.14×10^-73^ | 0.356 [0.333, 0.379] | 2.37×10^-201^ |
| Continuous^b^ | 0.021 [0.016, 0.025] | 1.59×10^-19^ | 0.009 [0.007, 0.01] | 6.20×10^-36^ | 0.105 [0.096, 0.113] | 5.63×10^-122^ |
| **Age**^c^ |  |  |  |  |  |  |
| ≥65 years | -0.079 [-0.091, -0.067] | 1.64×10^-38^ | 0.019 [0.015, 0.022] | 6.74×10^-26^ | 0.027 [0.004, 0.05] | 0.021 |
| Continuous | -0.006 [-0.007, -0.005] | 6.10×10^-69^ | 0.001 [0.001, 0.002] | 8.27×10^-45^ | -0.001 [-0.003, -1.7×10^-5^] | 0.047 |
| **Race/Ethnicity** |  |  |  |  |  |  |
| Mixed | 0.137 [0.071, 0.203] | 4.75×10^-5^ | -0.018 [-0.037, 0.002] | 0.077 | -0.063 [-0.19, 0.063] | 0.328 |
| Asian | 0.288 [0.25, 0.325] | 1.42×10^-50^ | -0.012 [-0.023, -8.44×10^-4^] | 0.035 | -0.199 [-0.272, -0.127] | 6.11×10^-8^ |
| Black | 0.192 [0.146, 0.237] | 1.38×10^-16^ | -0.097 [-0.11, -0.083] | 3.82×10^-45^ | -1.288 [-1.375, -1.201] | 3.66×10^-184^ |
| Other | 0.187 [0.135, 0.24] | 2.83×10^-12^ | -0.049 [-0.064, -0.033] | 8.33×10^-10^ | -0.36 [-0.46, -0.259] | 2.50×10^-12^ |
| **Alcohol drinking** |  |  |  |  |  |  |
| Former drinker | -0.036 [-0.086, 0.015] | 0.168 | -0.011 [-0.026, 0.004] | 0.133 | -0.033 [-0.13, 0.064] | 0.503 |
| Occasional | 0.012 [-0.036, 0.061] | 0.622 | -0.005 [-0.019, 0.01] | 0.531 | 0.054 [-0.039, 0.147] | 0.254 |
| 1-3 drink/month | -0.03 [-0.079, 0.018] | 0.215 | -0.006 [-0.02, 0.009] | 0.439 | -0.095 [-0.187, -0.003] | 0.044 |
| 1-2 drink/week | -0.083 [-0.13, -0.037] | 3.89×10^-4^ | -0.016 [-0.03, -0.003] | 0.019 | -0.237 [-0.325, -0.148] | 1.52×10^-7^ |
| 3-4 drink/week | -0.128 [-0.175, -0.082] | 4.64×10^-8^ | -0.025 [-0.038, -0.011] | 4.14×10^-4^ | -0.431 [-0.519, -0.343] | 1.03×10^-21^ |
| Daily | -0.124 [-0.169, -0.078] | 1.35×10^-7^ | -0.01 [-0.023, 0.004] | 0.159 | -0.401 [-0.489, -0.313] | 4.35×10^-19^ |
| **Body mass index** |  |  |  |  |  |  |
| < 18.5 | -0.022 [-0.119, 0.075] | 0.657 | 0.052 [0.023, 0.081] | 4.09×10^-4^ | 0.817 [0.631, 1.004] | 9.70×10^-18^ |
| 25 to <30 | 0.082 [0.069, 0.094] | 6.24×10^-38^ | 0.018 [0.014, 0.021] | 7.08×10^-21^ | -0.059 [-0.083, -0.036] | 1.13×10^-6^ |
| 30 to <35 | 0.19 [0.175, 0.205] | 1.99×10^-138^ | 0.046 [0.042, 0.051] | 6.60×10^-94^ | 0.014 [-0.015, 0.042] | 0.34 |
| >35 | 0.253 [0.231, 0.276] | 1.30×10^-110^ | 0.076 [0.07, 0.083] | 1.54×10^-113^ | 0.265 [0.222, 0.308] | 4.59×10^-34^ |
| Continuous | 0.018 [0.017, 0.019] | 4.48×10^-196^ | 0.005 [0.005, 0.005] | 4.80×10^-169^ | 0.011 [0.009, 0.014] | 7.19×10^-23^ |
| **Diabetes** | 0.036 [0.017, 0.056] | 2.42×10^-4^ | 0.005 [-5.69×10^-4^, 0.011] | 0.077 | 0.21 [0.173, 0.247] | 3.05×10^-28^ |
| **Hypertension** | -0.018 [-0.029, -0.007] | 0.001 | 0.022 [0.019, 0.025] | 3.58×10^-39^ | 0.224 [0.203, 0.246] | 1.62×10^-94^ |
| **Hypercholesterolemia** | 0.046 [0.033, 0.059] | 7.34×10^-12^ | 0.013 [0.009, 0.017] | 1.05×10^-10^ | 0.126 [0.101, 0.152] | 7.28×10^-23^ |

^a^P-values from multivariable linear regression models adjusted for all other variables in this table. Unless specified otherwise, we adjusted for age, age squared, race/ethnicity, alcohol consumption, body mass index (continuous variable), diabetes, hypertension, and hypercholesterolemia. Except for continuous variable rows, the reference group for categorical variables were no mLOY, White, never drinker, 18.5 ≤ body mass index < 25, no diabetes, no hypertension, no hypercholesteroemia.
^b^The continuous variable reports the coefficient when mLRR was standardized.
^c^The continuous variable reports the coefficient for age without the squared term.

**Table S12. Linear regression for erythrocyte, leukocyte, and platelet counts by mLOY status in never smokers**

|  | Leukocyte Count (×10^9^ cells/L) | | Erythrocyte Count (×10^12^ cells/L) | | Thrombocyte Count (×10^9^ cells/L) | |
| --- | --- | --- | --- | --- | --- | --- |
|  | Estimate (95% CI) | *P*^a^ | Estimate (95% CI) | *P* | Estimate (95% CI) | *P* |
| **mLOY** |  |  |  |  |  |  |
| Categorical | 0.145 [0.114, 0.176] | 9.82×10^-20^ | -0.007 [-0.014, -2.63×10^-4^] | 0.042 | 5.021 [4.017, 6.024] | 1.13×10^-22^ |
| Continuous^b^ | 0.136 [0.125, 0.147] | 5.99×10^-126^ | -0.013 [-0.015, -0.011] | 2.79×10^-32^ | 2.651 [2.321, 2.982] | 1.23×10^-55^ |
| **Age**^c^ |  |  |  |  |  |  |
| ≥65 years | 0.204 [0.174, 0.235] | 1.74×10^-39^ | -0.068 [-0.075, -0.062] | 9.57×10^-93^ | -11.754 [-12.734, -10.774] | 8.14×10^-122^ |
| Continuous | 0.013 [0.012, 0.015] | 1.36×10^-73^ | -0.005 [-0.006, -0.005] | 5.83×10^-249^ | -0.741 [-0.786, -0.695] | 6.27×10^-221^ |
| **Race/Ethnicity** |  |  |  |  |  |  |
| Mixed | 0.017 [-0.14, 0.175] | 0.831 | 0.071 [0.037, 0.104] | 4.16×10^-5^ | -3.433 [-8.495, 1.629] | 0.184 |
| Asian | 0.389 [0.327, 0.451] | 4.50×10^-35^ | 0.181 [0.168, 0.195] | 2.03×10^-159^ | 3.012 [1.031, 4.993] | 0.003 |
| Black | -1.357 [-1.438, -1.276] | 9.90×10^-235^ | 0.13 [0.113, 0.148] | 3.94×10^-49^ | -29.487 [-32.09, -26.883] | 6.77×10^-109^ |
| Other | -0.249 [-0.365, -0.134] | 2.37×10^-5^ | 0.183 [0.159, 0.208] | 8.87×10^-48^ | -7.957 [-11.668, -4.246] | 2.64×10^-5^ |
| **Alcohol drinking** |  |  |  |  |  |  |
| Former drinker | 0.074 [-0.009, 0.157] | 0.082 | -0.062 [-0.08, -0.045] | 7.01×10^-12^ | -1.056 [-3.729, 1.618] | 0.439 |
| Occasional | -0.01 [-0.075, 0.054] | 0.757 | -0.01 [-0.024, 0.003] | 0.143 | -2.84 [-4.911, -0.77] | 0.007 |
| 1-3 drink/month | -0.013 [-0.076, 0.05] | 0.69 | -0.009 [-0.023, 0.004] | 0.179 | -1.247 [-3.269, 0.776] | 0.227 |
| 1-2 drink/week | -0.078 [-0.135, -0.02] | 0.008 | -0.044 [-0.056, -0.031] | 3.11×10^-12^ | -1.82 [-3.659, 0.019] | 0.052 |
| 3-4 drink/week | -0.184 [-0.242, -0.127] | 3.81×10^-10^ | -0.078 [-0.09, -0.065] | 5.40×10^-35^ | -2.578 [-4.432, -0.725] | 0.006 |
| Daily | -0.222 [-0.281, -0.163] | 1.81×10^-13^ | -0.107 [-0.12, -0.095] | 4.36×10^-62^ | -2.502 [-4.4, -0.604] | 0.01 |
| **Body mass index** |  |  |  |  |  |  |
| < 18.5 | 0.268 [0.016, 0.519] | 0.037 | -0.238 [-0.292, -0.185] | 3.61×10^-18^ | -5.183 [-13.242, 2.877] | 0.208 |
| 25 to <30 | 0.282 [0.257, 0.308] | 1.05×10^-105^ | 0.096 [0.091, 0.101] | 2.01×10^-263^ | 0.608 [-0.204, 1.42] | 0.142 |
| 30 to <35 | 0.592 [0.559, 0.625] | 4.51×10^-268^ | 0.139 [0.132, 0.146] | < 5×10^-324^ | -0.429 [-1.489, 0.631] | 0.427 |
| >35 | 1.069 [1.016, 1.122] | < 5×10^-324^ | 0.134 [0.122, 0.145] | 3.19×10^-117^ | 0.807 [-0.901, 2.514] | 0.355 |
| Continuous | 0.07 [0.067, 0.072] | < 5×10^-324^ | 0.012 [0.012, 0.013] | < 5×10^-324^ | 0.064 [-0.022, 0.15] | 0.146 |
| **Diabetes** | 0.297 [0.245, 0.35] | 8.99×10^-29^ | -0.113 [-0.124, -0.102] | 8.18×10^-87^ | -2.477 [-4.159, -0.796] | 0.004 |
| **Hypertension** | 0.221 [0.195, 0.247] | 3.98×10^-61^ | 0.014 [0.009, 0.02] | 5.33×10^-7^ | 2.613 [1.77, 3.456] | 1.26×10^-9^ |
| **Hypercholesterolemia** | 0.126 [0.092, 0.159] | 1.73×10^-13^ | -0.015 [-0.023, -0.008] | 2.29×10^-5^ | -2.686 [-3.759, -1.613] | 9.23×10^-7^ |

^a^P-values from multivariable linear regression models adjusted for all other variables in this table. Unless specified otherwise, we adjusted for age, age squared, race/ethnicity, alcohol consumption, body mass index (continuous variable), diabetes, hypertension, and hypercholesterolemia. Except for continuous variable rows, the reference group for categorical variables were no mLOY, White, never drinker, 18.5 ≤ body mass index < 25, no diabetes, no hypertension, no hypercholesteroemia.
^b^The continuous variable reports the coefficient when mLRR was standardized.
^c^The continuous variable reports the coefficient for age without the squared term.

**Table S13. Linear regression for lymphocyte, monocyte and neutrophil counts by mLOY status in never smokers**

|  | Lymphocyte Count (×10^9^ cells/L) | | Monocyte Count (×10^9^ cells/L) | | Neutrophil Count (×10^9^ cells/L) | |
| --- | --- | --- | --- | --- | --- | --- |
|  | Estimate (95% CI) | *P*^a^ | Estimate (95% CI) | *P* | Estimate (95% CI) | *P* |
| **mLOY** |  |  |  |  |  |  |
| Categorical | -0.005 [-0.02, 0.01] | 0.516 | 0.019 [0.015, 0.023] | 7.18×10^-22^ | 0.125 [0.101, 0.148] | 8.08×10^-25^ |
| Continuous^b^ | 0.021 [0.016, 0.025] | 1.59×10^-19^ | 0.009 [0.007, 0.01] | 6.20×10^-36^ | 0.105 [0.096, 0.113] | 5.63×10^-122^ |
| **Age**^c^ |  |  |  |  |  |  |
| ≥65 years | -0.009 [-0.023, 0.005] | 0.218 | 0.028 [0.024, 0.031] | 1.11×10^-47^ | 0.186 [0.162, 0.209] | 1.14×10^-55^ |
| Continuous | -6.48×10^-4^ [-0.001, 2.29×10^-5^] | 0.058 | 0.002 [0.002, 0.002] | 1.31×10^-120^ | 0.012 [0.011, 0.013] | 3.48×10^-101^ |
| **Race/Ethnicity** |  |  |  |  |  |  |
| Mixed | 0.124 [0.049, 0.198] | 0.001 | -0.015 [-0.034, 0.004] | 0.129 | -0.104 [-0.223, 0.016] | 0.089 |
| Asian | 0.352 [0.323, 0.381] | 2.01×10^-123^ | -0.003 [-0.01, 0.005] | 0.502 | -0.019 [-0.066, 0.027] | 0.416 |
| Black | 0.128 [0.089, 0.166] | 7.31×10^-11^ | -0.105 [-0.115, -0.095] | 1.17×10^-95^ | -1.364 [-1.426, -1.302] | < 5×10^-324^ |
| Other | 0.179 [0.124, 0.233] | 1.40×10^-10^ | -0.042 [-0.056, -0.028] | 4.21×10^-9^ | -0.393 [-0.481, -0.306] | 1.40×10^-18^ |
| **Alcohol drinking** |  |  |  |  |  |  |
| Former drinker | -0.007 [-0.047, 0.032] | 0.718 | -0.006 [-0.016, 0.004] | 0.259 | 0.059 [-0.004, 0.122] | 0.067 |
| Occasional | 0.003 [-0.028, 0.033] | 0.864 | -0.003 [-0.011, 0.005] | 0.496 | -0.006 [-0.055, 0.043] | 0.8 |
| 1-3 drink/month | 0.018 [-0.012, 0.048] | 0.232 | 4.2×10^-4^ [-0.007, 0.008] | 0.915 | -0.031 [-0.079, 0.017] | 0.202 |
| 1-2 drink/week | 0.028 [7.73×10^-4^, 0.055] | 0.044 | -0.007 [-0.014, 1.65×10^-4^] | 0.056 | -0.094 [-0.137, -0.05] | 2.40×10^-5^ |
| 3-4 drink/week | 0.016 [-0.012, 0.043] | 0.262 | -0.01 [-0.017, -0.003] | 0.006 | -0.183 [-0.227, -0.139] | 2.60×10^-16^ |
| Daily | -0.017 [-0.045, 0.011] | 0.229 | -0.008 [-0.016, -0.001] | 0.024 | -0.191 [-0.235, -0.146] | 8.55×10^-17^ |
| **Body mass index** |  |  |  |  |  |  |
| < 18.5 | -0.276 [-0.394, -0.158] | 4.91×10^-6^ | -0.012 [-0.043, 0.018] | 0.429 | 0.17 [-0.021, 0.362] | 0.08 |
| 25 to <30 | 0.128 [0.116, 0.14] | 1.74×10^-98^ | 0.023 [0.02, 0.026] | 3.06×10^-48^ | 0.122 [0.102, 0.141] | 2.91×10^-35^ |
| 30 to <35 | 0.253 [0.238, 0.269] | 6.93×10^-223^ | 0.056 [0.052, 0.06] | 1.38×10^-164^ | 0.261 [0.236, 0.286] | 1.59×10^-92^ |
| >35 | 0.337 [0.312, 0.362] | 1.24×10^-152^ | 0.091 [0.085, 0.098] | 2.30×10^-166^ | 0.604 [0.564, 0.644] | 6.49×10^-188^ |
| Continuous | 0.025 [0.024, 0.027] | < 5×10^-324^ | 0.006 [0.006, 0.006] | 1.57×10^-291^ | 0.036 [0.034, 0.038] | 1.94×10^-265^ |
| **Diabetes** | 0.05 [0.025, 0.075] | 7.99×10^-5^ | 0.008 [0.001, 0.014] | 0.02 | 0.218 [0.178, 0.257] | 7.16×10^-27^ |
| **Hypertension** | -0.009 [-0.021, 0.004] | 0.162 | 0.018 [0.015, 0.021] | 9.94×10^-28^ | 0.21 [0.191, 0.23] | 4.74×10^-95^ |
| **Hypercholesterolemia** | 0.051 [0.035, 0.066] | 3.66×10^-10^ | 0.014 [0.009, 0.018] | 7.65×10^-11^ | 0.058 [0.033, 0.083] | 7.30×10^-6^ |

^a^P-values from multivariable linear regression models adjusted for all other variables in this table. Unless specified otherwise, we adjusted for age, age squared, race/ethnicity, alcohol consumption, body mass index (continuous variable), diabetes, hypertension, and hypercholesterolemia. Except for continuous variable rows, the reference group for categorical variables were no mLOY, White, never drinker, 18.5 ≤ body mass index < 25, no diabetes, no hypertension, no hypercholesteroemia.
^b^The continuous variable reports the coefficient when mLRR was standardized.
^c^The continuous variable reports the coefficient for age without the squared term.

**Table S14. Associations between neutrophil-lymphocyte ratio (NLR) and mLOY in ever and never smokers**

|  | NLR^a^ | | | |
| --- | --- | --- | --- | --- |
|  | Ever |  | Never |  |
|  | Estimate (95% CI) | *P*^b^ | Estimate (95% CI) | *P* |
| **mLOY** |  |  |  |  |
| Categorical | 0.092 [0.072, 0.111] | 1.21×10^-19^ | 0.038 [0.013, 0.064] | 0.003 |
| Continuous^c^ | 0.042 [0.035, 0.05] | 9.34×10^-29^ | 0.038 [0.027, 0.048] | 1.87×10^-12^ |
| **Age** |  |  |  |  |
| ≥65 years | 0.174 [0.154, 0.193] | 7.53×10^-68^ | 0.2 [0.176, 0.225] | 1.79×10^-57^ |
| Continuous^d^ | 0.01 [0.009, 0.012] | 6.32×10^-79^ | 0.012 [0.011, 0.013] | 1.94×10^-88^ |
| **Race/Ethnicity** |  |  |  |  |
| Mixed | -0.2 [-0.309, -0.092] | 2.99×10^-4^ | -0.226 [-0.353, -0.099] | 4.80×10^-4^ |
| Asian | -0.413 [-0.475, -0.351] | 6.18×10^-39^ | -0.38 [-0.43, -0.331] | 9.39×10^-51^ |
| Black | -0.843 [-0.918, -0.768] | 7.80×10^-108^ | -0.916 [-0.982, -0.851] | 2.56×10^-165^ |
| Other | -0.387 [-0.474, -0.301] | 1.70×10^-18^ | -0.462 [-0.555, -0.369] | 2.40×10^-22^ |
| **Alcohol drinking** |  |  |  |  |
| Former drinker | 0.049 [-0.034, 0.133] | 0.244 | 0.066 [-9.1×10^-4^, 0.133] | 0.053 |
| Occasional | 0.025 [-0.054, 0.105] | 0.531 | -0.003 [-0.055, 0.049] | 0.913 |
| 1-3 drink/month | -0.028 [-0.107, 0.052] | 0.494 | -0.038 [-0.088, 0.013] | 0.145 |
| 1-2 drink/week | -0.036 [-0.112, 0.04] | 0.351 | -0.114 [-0.16, -0.068] | 1.37×10^-6^ |
| 3-4 drink/week | -0.087 [-0.163, -0.012] | 0.024 | -0.163 [-0.209, -0.116] | 6.61×10^-12^ |
| Daily | -0.081 [-0.157, -0.006] | 0.035 | -0.141 [-0.188, -0.093] | 6.89×10^-9^ |
| **Body mass index** |  |  |  |  |
| < 18.5 | 0.606 [0.436, 0.776] | 3.16×10^-12^ | 0.856 [0.654, 1.059] | 1.16×10^-16^ |
| 25 to <30 | -0.173 [-0.195, -0.151] | 3.56×10^-54^ | -0.135 [-0.156, -0.115] | 9.68×10^-39^ |
| 30 to <35 | -0.273 [-0.299, -0.247] | 2.30×10^-93^ | -0.203 [-0.23, -0.176] | 1.22×10^-50^ |
| >35 | -0.224 [-0.263, -0.185] | 1.69×10^-29^ | -0.174 [-0.217, -0.131] | 1.61×10^-15^ |
| Continuous | -0.02 [-0.022, -0.018] | 7.70×10^-89^ | -0.017 [-0.019, -0.014] | 1.30×10^-51^ |
| **Diabetes** | 0.098 [0.066, 0.13] | 2.40×10^-9^ | 0.119 [0.077, 0.161] | 3.04×10^-8^ |
| **Hypertension** | 0.172 [0.153, 0.19] | 2.70×10^-75^ | 0.172 [0.15, 0.193] | 7.90×10^-57^ |
| **Hypercholesterolemia** | 0.006 [-0.016, 0.027] | 0.602 | -0.035 [-0.062, -0.008] | 0.011 |

^a^Mean and standard deviation in subjects without mLOY: ever (2.437, 1.234); never (2.407, 1.398)
^b^P-values from multivariable linear regression models adjusted for all other variables in this table. Unless specified otherwise, we adjusted for age, age squared, race/ethnicity, alcohol consumption, body mass index (continuous variable), diabetes, hypertension, and hypercholesterolemia. Except for continuous variable rows, the reference group for categorical variables were no mLOY, White, 18.5 ≤ body mass index < 25, no diabetes, no hypertension, no hypercholesteroemia.
^c^The continuous variable reports the coefficient when mLRR was standardized.
^d^The continuous variable reports the coefficient for age without the squared term.

**Table S15. Associations between thrombocyte-lymphocyte ratio (TLR) and mLOY in ever and never smokers**

|  | TLR^a^ | | | |
| --- | --- | --- | --- | --- |
|  | Ever | | Never |  |
|  | Estimate (95% CI) | *P*^b^ | Estimate (95% CI) | *P* |
| **mLOY** |  |  |  |  |
| Categorical | -1.514 [-2.406, -0.621] | 8.86×10^-4^ | 1.51 [0.319, 2.7] | 0.013 |
| Continuous^c^ | 0.699 [0.362, 1.036] | 4.84×10^-5^ | 2.081 [1.585, 2.577] | 2.00×10^-16^ |
| **Age** |  |  |  |  |
| ≥65 years | 1.048 [0.167, 1.929] | 0.02 | -2.884 [-4.044, -1.725] | 1.09×10^-6^ |
| Continuous^d^ | 0.098 [0.049, 0.147] | 9.42×10^-5^ | -0.212 [-0.266, -0.158] | 1.58×10^-14^ |
| **Race/Ethnicity** |  |  |  |  |
| Mixed | -8.469 [-13.369, -3.569] | 7.05×10^-4^ | -11.185 [-17.188, -5.182] | 2.60×10^-4^ |
| Asian | -19.617 [-22.413, -16.82] | 5.71×10^-43^ | -19.553 [-21.903, -17.204] | 1.00×10^-59^ |
| Black | -19.902 [-23.276, -16.527] | 6.90×10^-31^ | -25.133 [-28.225, -22.042] | 4.42×10^-57^ |
| Other | -14.165 [-18.066, -10.264] | 1.11×10^-12^ | -19.374 [-23.769, -14.978] | 5.79×10^-18^ |
| **Alcohol drinking** |  |  |  |  |
| Former drinker | 2.103 [-1.653, 5.859] | 0.272 | 0.04 [-3.129, 3.209] | 0.98 |
| Occasional | -0.422 [-4.015, 3.171] | 0.818 | -1.604 [-4.059, 0.852] | 0.2 |
| 1-3 drink/month | 0.142 [-3.434, 3.719] | 0.938 | -2.091 [-4.49, 0.307] | 0.087 |
| 1-2 drink/week | 2.982 [-0.44, 6.404] | 0.088 | -4.063 [-6.243, -1.882] | 2.61×10^-4^ |
| 3-4 drink/week | 4.598 [1.178, 8.019] | 0.008 | -4.145 [-6.343, -1.947] | 2.19×10^-4^ |
| Daily | 4.368 [0.958, 7.779] | 0.012 | -2.17 [-4.421, 0.081] | 0.059 |
| **Body mass index** |  |  |  |  |
| < 18.5 | 16.839 [9.308, 24.369] | 1.17×10^-5^ | 31.092 [21.505, 40.679] | 2.07×10^-10^ |
| 25 to <30 | -10.645 [-11.611, -9.679] | 2.98×10^-103^ | -12.488 [-13.452, -11.524] | 1.03×10^-141^ |
| 30 to <35 | -20.447 [-21.6, -19.295] | 2.48×10^-263^ | -21.241 [-22.499, -19.983] | 1.02×10^-238^ |
| >35 | -25.626 [-27.348, -23.904] | 3.02×10^-186^ | -28.436 [-30.463, -26.409] | 7.57×10^-166^ |
| Continuous | -1.866 [-1.954, -1.778] | < 5×10^-324^ | -2.11 [-2.212, -2.008] | < 5×10^-324^ |
| **Diabetes** | -2.424 [-3.87, -0.978] | 0.001 | -1.363 [-3.357, 0.631] | 0.18 |
| **Hypertension** | 3.912 [3.086, 4.738] | 1.64×10^-20^ | 3.434 [2.434, 4.433] | 1.68×10^-11^ |
| **Hypercholesterolemia** | -4.266 [-5.242, -3.291] | 1.04×10^-17^ | -5.666 [-6.939, -4.394] | 2.62×10^-18^ |

^a^Mean and standard deviation in subjects without mLOY: ever (134.653, 58.329); never (141.397, 66.197)
^b^P-values from multivariable linear regression models adjusted for all other variables in this table. Unless specified otherwise, we adjusted for age, age squared, race/ethnicity, alcohol consumption, body mass index (continuous variable), diabetes, hypertension, and hypercholesterolemia. Except for continuous variable rows, the reference group for categorical variables were no mLOY, White, 18.5 ≤ body mass index < 25, no diabetes, no hypertension, no hypercholesteroemia.
^c^The continuous variable reports the coefficient when mLRR was standardized.
^d^The continuous variable reports the coefficient for age without the squared term.

**Table S16. Linear regression for erythrocyte, leukocyte, and platelet counts by mLOY status in subjects less than 65 years old**

|  | Leukocyte Count (×10^9^ cells/L) | | Erythrocyte Count (×10^12^ cells/L) | | Thrombocyte Count (×10^9^ cells/L) | |
| --- | --- | --- | --- | --- | --- | --- |
|  | Estimate (95% CI) | *P*^a^ | Estimate (95% CI) | *P* | Estimate (95% CI) | *P* |
| **mLOY** |  |  |  |  |  |  |
| Categorical | 0.167 [0.129, 0.205] | 1.03×10^-17^ | -0.02 [-0.028, -0.012] | 1.32×10^-6^ | 5.293 [4.134, 6.451] | 3.66×10^-19^ |
| Continuous^b^ | 0.061 [0.048, 0.075] | 3.20×10^-19^ | -0.013 [-0.016, -0.01] | 2.05×10^-20^ | 2.233 [1.828, 2.639] | 4.10×10^-27^ |
| **Race/Ethnicity** |  |  |  |  |  |  |
| Mixed | -0.213 [-0.596, 0.169] | 0.274 | 0.058 [-0.022, 0.138] | 0.153 | 0.07 [-11.531, 11.671] | 0.991 |
| Asian | 0.188 [0.047, 0.33] | 0.009 | 0.099 [0.07, 0.129] | 4.16×10^-11^ | 0.21 [-4.074, 4.493] | 0.924 |
| Black | -1.477 [-1.695, -1.26] | 3.30×10^-40^ | 0.059 [0.013, 0.105] | 0.011 | -21.218 [-27.828, -14.609] | 3.17×10^-10^ |
| Other | -0.299 [-0.593, -0.004] | 0.047 | 0.191 [0.129, 0.252] | 1.18×10^-9^ | -13.02 [-21.951, -4.089] | 0.004 |
| **Smoking status** |  |  |  |  |  |  |
| Current smoker | 0.197 [0.157, 0.237] | 7.57×10^-22^ | -0.029 [-0.037, -0.02] | 2.11×10^-11^ | 5.546 [4.332, 6.761] | 3.69×10^-19^ |
| Former smoker | 1.327 [1.258, 1.397] | 8.60×10^-299^ | -0.048 [-0.063, -0.034] | 8.47×10^-11^ | 13.457 [11.349, 15.564] | 7.21×10^-36^ |
| **Alcohol drinking** |  |  |  |  |  |  |
| Former drinker | 0.082 [-0.064, 0.228] | 0.27 | -0.046 [-0.077, -0.016] | 0.003 | 1.337 [-3.092, 5.766] | 0.554 |
| Occasional | 0.043 [-0.082, 0.169] | 0.498 | -0.004 [-0.03, 0.022] | 0.765 | -2.158 [-5.973, 1.657] | 0.268 |
| 1-3 drink/month | 0.005 [-0.123, 0.132] | 0.941 | 0.007 [-0.02, 0.033] | 0.63 | -1.621 [-5.491, 2.248] | 0.411 |
| 1-2 drink/week | -0.066 [-0.181, 0.049] | 0.258 | -0.022 [-0.046, 0.002] | 0.074 | -1.487 [-4.967, 1.994] | 0.402 |
| 3-4 drink/week | -0.201 [-0.316, -0.086] | 6.20×10^-4^ | -0.055 [-0.079, -0.031] | 6.80×10^-6^ | -2.324 [-5.812, 1.163] | 0.191 |
| Daily | -0.293 [-0.408, -0.179] | 5.11×10^-7^ | -0.091 [-0.115, -0.067] | 8.88×10^-14^ | -2.592 [-6.062, 0.877] | 0.143 |
| **Body mass index** |  |  |  |  |  |  |
| < 18.5 | 0.23 [-0.229, 0.689] | 0.326 | -0.212 [-0.308, -0.116] | 1.52×10^-5^ | 3.704 [-10.276, 17.684] | 0.604 |
| 25 to <30 | 0.195 [0.148, 0.242] | 2.81×10^-16^ | 0.091 [0.082, 0.101] | 1.85×10^-74^ | -5.494 [-6.916, -4.072] | 3.76×10^-14^ |
| 30 to <35 | 0.448 [0.39, 0.506] | 1.68×10^-51^ | 0.128 [0.116, 0.14] | 1.37×10^-93^ | -9.477 [-11.245, -7.709] | 8.83×10^-26^ |
| >35 | 0.759 [0.665, 0.854] | 2.09×10^-55^ | 0.085 [0.065, 0.105] | 4.83×10^-17^ | -10.779 [-13.664, -7.895] | 2.46×10^-13^ |
| Continuous | 0.053 [0.048, 0.058] | 1.16×10^-97^ | 0.01 [0.009, 0.011] | 2.10×10^-84^ | -0.82 [-0.971, -0.669] | 1.64×10^-26^ |
| **Diabetes** | 0.236 [0.17, 0.301] | 2.05×10^-12^ | -0.119 [-0.132, -0.105] | 2.73×10^-64^ | -3.454 [-5.446, -1.462] | 6.78×10^-4^ |
| **Hypertension** | 0.24 [0.201, 0.28] | 1.39×10^-32^ | 0.002 [-0.006, 0.011] | 0.585 | 2.933 [1.733, 4.132] | 1.66×10^-6^ |
| **Hypercholesterolemia** | 0.125 [0.082, 0.169] | 1.42×10^-8^ | -0.022 [-0.031, -0.013] | 2.18×10^-6^ | -3.785 [-5.098, -2.472] | 1.62×10^-8^ |

^a^P-values from multivariable linear regression models adjusted for all other variables in this table. Unless specified otherwise, we adjusted for race/ethnicity, alcohol consumption, body mass index (continuous variable), diabetes, hypertension, and hypercholesterolemia. Except for continuous variable rows, the reference group for categorical variables were no mLOY, White, never drinker, 18.5 ≤ body mass index < 25, no diabetes, no hypertension, no hypercholesteroemia.
^b^The continuous variable reports the coefficient when mLRR was standardized.

**Table S17. Linear regression for lymphocyte, monocyte and neutrophil counts by mLOY status in subjects less than 65 years old**

|  | Lymphocyte Count (×10^9^ cells/L) | | Monocyte Count (×10^9^ cells/L) | | Neutrophil Count (×10^9^ cells/L) | |
| --- | --- | --- | --- | --- | --- | --- |
|  | Estimate (95% CI) | *P*^a^ | Estimate (95% CI) | *P* | Estimate (95% CI) | *P* |
| **mLOY** |  |  |  |  |  |  |
| Categorical | -0.006 [-0.026, 0.013] | 0.526 | 0.019 [0.014, 0.024] | 7.07×10^-13^ | 0.148 [0.119, 0.177] | 9.50×10^-24^ |
| Continuous^b^ | -0.002 [-0.009, 0.005] | 0.561 | 0.006 [0.004, 0.008] | 5.89×10^-12^ | 0.058 [0.048, 0.068] | 1.77×10^-29^ |
| **Race/Ethnicity** |  |  |  |  |  |  |
| Mixed | 0.085 [-0.11, 0.279] | 0.394 | -0.016 [-0.067, 0.035] | 0.542 | -0.32 [-0.608, -0.032] | 0.029 |
| Asian | 0.314 [0.242, 0.386] | 1.14×10^-17^ | -0.008 [-0.027, 0.011] | 0.391 | -0.184 [-0.291, -0.078] | 6.95×10^-4^ |
| Black | 0.142 [0.03, 0.253] | 0.013 | -0.122 [-0.152, -0.093] | 1.60×10^-16^ | -1.488 [-1.653, -1.323] | 6.31×10^-70^ |
| Other | 0.202 [0.052, 0.351] | 0.008 | -0.057 [-0.097, -0.018] | 0.004 | -0.462 [-0.684, -0.241] | 4.36×10^-5^ |
| **Smoking status** |  |  |  |  |  |  |
| Current smoker | 0.036 [0.015, 0.056] | 5.87×10^-4^ | 0.017 [0.011, 0.022] | 7.61×10^-10^ | 0.129 [0.099, 0.159] | 6.59×10^-17^ |
| Former smoker | 0.334 [0.298, 0.369] | 4.73×10^-76^ | 0.059 [0.05, 0.068] | 8.45×10^-36^ | 0.889 [0.836, 0.942] | 1.48×10^-237^ |
| **Alcohol drinking** |  |  |  |  |  |  |
| Former drinker | 0.028 [-0.046, 0.102] | 0.46 | -0.005 [-0.024, 0.015] | 0.626 | 0.064 [-0.046, 0.174] | 0.255 |
| Occasional | 0.048 [-0.016, 0.112] | 0.144 | -0.014 [-0.031, 0.003] | 0.102 | 0.023 [-0.072, 0.117] | 0.64 |
| 1-3 drink/month | 0.039 [-0.026, 0.104] | 0.237 | -6.31×10^-4^ [-0.018, 0.016] | 0.942 | -0.027 [-0.123, 0.069] | 0.583 |
| 1-2 drink/week | 0.035 [-0.023, 0.094] | 0.238 | -0.014 [-0.029, 0.002] | 0.08 | -0.081 [-0.168, 0.005] | 0.065 |
| 3-4 drink/week | 0.019 [-0.039, 0.078] | 0.516 | -0.014 [-0.03, 0.001] | 0.068 | -0.197 [-0.284, -0.111] | 8.09×10^-6^ |
| Daily | -0.014 [-0.072, 0.044] | 0.643 | -0.016 [-0.031, -5.73×10^-4^] | 0.042 | -0.249 [-0.335, -0.163] | 1.55×10^-8^ |
| **Body mass index** |  |  |  |  |  |  |
| < 18.5 | -0.262 [-0.494, -0.03] | 0.027 | 0.033 [-0.028, 0.094] | 0.291 | 0.495 [0.149, 0.842] | 0.005 |
| 25 to <30 | 0.121 [0.098, 0.145] | 9.36×10^-24^ | 0.027 [0.021, 0.033] | 2.56×10^-17^ | 0.036 [8.49×10^-4^, 0.071] | 0.045 |
| 30 to <35 | 0.244 [0.214, 0.273] | 3.38×10^-59^ | 0.055 [0.047, 0.062] | 1.03×10^-43^ | 0.13 [0.086, 0.174] | 6.78×10^-9^ |
| >35 | 0.306 [0.258, 0.354] | 7.45×10^-36^ | 0.087 [0.075, 0.1] | 2.96×10^-42^ | 0.334 [0.262, 0.405] | 6.53×10^-20^ |
| Continuous | 0.025 [0.022, 0.027] | 3.04×10^-82^ | 0.006 [0.005, 0.007] | 1.65×10^-72^ | 0.02 [0.016, 0.024] | 1.03×10^-25^ |
| **Diabetes** | 0.03 [-0.004, 0.063] | 0.08 | 0.004 [-0.004, 0.013] | 0.322 | 0.191 [0.141, 0.24] | 4.36×10^-14^ |
| **Hypertension** | -0.023 [-0.044, -0.003] | 0.022 | 0.023 [0.018, 0.029] | 4.55×10^-18^ | 0.235 [0.205, 0.265] | 1.22×10^-53^ |
| **Hypercholesterolemia** | 0.041 [0.019, 0.063] | 3.11×10^-4^ | 0.007 [0.001, 0.013] | 0.019 | 0.076 [0.044, 0.109] | 4.79×10^-6^ |

^a^P-values from multivariable linear regression models adjusted for all other variables in this table. Unless specified otherwise, we adjusted for race/ethnicity, alcohol consumption, body mass index (continuous variable), diabetes, hypertension, and hypercholesterolemia. Except for continuous variable rows, the reference group for categorical variables were no mLOY, White, never drinker, 18.5 ≤ body mass index < 25, no diabetes, no hypertension, no hypercholesteroemia.
^b^The continuous variable reports the coefficient when mLRR was standardized.

**Table S18. Linear regression for erythrocyte, leukocyte, and platelet counts by mLOY status in subjects more than 65 years old**

|  | Leukocyte Count (×10^9^ cells/L) | | Erythrocyte Count (×10^12^ cells/L) | | Thrombocyte Count (×10^9^ cells/L) | |
| --- | --- | --- | --- | --- | --- | --- |
|  | Estimate (95% CI) | *P*^a^ | Estimate (95% CI) | *P* | Estimate (95% CI) | *P* |
| **mLOY** |  |  |  |  |  |  |
| Categorical | 0.243 [0.218, 0.268] | 3.42×10^-81^ | -0.006 [-0.012, -0.001] | 0.017 | 5.435 [4.629, 6.24] | 6.88×10^-40^ |
| Continuous^b^ | 0.076 [0.065, 0.086] | 5.68×10^-47^ | -0.012 [-0.014, -0.01] | 2.29×10^-27^ | 1.413 [1.08, 1.747] | 1.01×10^-16^ |
| **Race/Ethnicity** |  |  |  |  |  |  |
| Mixed | 0.009 [-0.103, 0.122] | 0.869 | 0.076 [0.053, 0.1] | 2.83×10^-10^ | -1.693 [-5.322, 1.936] | 0.361 |
| Asian | 0.319 [0.266, 0.373] | 2.57×10^-31^ | 0.202 [0.191, 0.214] | 2.97×10^-266^ | 0.289 [-1.448, 2.026] | 0.744 |
| Black | -1.265 [-1.331, -1.198] | 2.49×10^-301^ | 0.109 [0.095, 0.123] | 2.09×10^-52^ | -25.363 [-27.517, -23.209] | 1.12×10^-117^ |
| Other | -0.215 [-0.301, -0.129] | 1.00×10^-6^ | 0.156 [0.138, 0.175] | 8.94×10^-64^ | -7.44 [-10.224, -4.656] | 1.63×10^-7^ |
| **Smoking status** |  |  |  |  |  |  |
| Current smoker | 0.143 [0.123, 0.162] | 1.32×10^-48^ | -0.039 [-0.043, -0.035] | 6.05×10^-83^ | 2.53 [1.92, 3.139] | 4.11×10^-16^ |
| Former smoker | 1.487 [1.461, 1.514] | < 5×10^-324^ | -0.053 [-0.059, -0.048] | 8.97×10^-80^ | 8.808 [7.967, 9.649] | 1.57×10^-93^ |
| **Alcohol drinking** |  |  |  |  |  |  |
| Former drinker | 0.024 [-0.046, 0.094] | 0.505 | -0.073 [-0.088, -0.058] | 4.57×10^-22^ | -1.615 [-3.876, 0.646] | 0.162 |
| Occasional | 0.031 [-0.03, 0.093] | 0.32 | -0.013 [-0.026, -2.98×10^-4^] | 0.045 | -2.054 [-4.039, -0.07] | 0.042 |
| 1-3 drink/month | -0.021 [-0.081, 0.039] | 0.492 | -0.017 [-0.03, -0.005] | 0.007 | -1.829 [-3.775, 0.117] | 0.065 |
| 1-2 drink/week | -0.099 [-0.155, -0.042] | 5.88×10^-4^ | -0.057 [-0.069, -0.045] | 4.78×10^-21^ | -2.105 [-3.921, -0.289] | 0.023 |
| 3-4 drink/week | -0.23 [-0.287, -0.174] | 1.26×10^-15^ | -0.093 [-0.105, -0.081] | 3.03×10^-53^ | -3.212 [-5.036, -1.388] | 5.59×10^-4^ |
| Daily | -0.271 [-0.328, -0.214] | 1.28×10^-20^ | -0.129 [-0.141, -0.117] | 5.27×10^-98^ | -3.558 [-5.398, -1.718] | 1.51×10^-4^ |
| **Body mass index** |  |  |  |  |  |  |
| < 18.5 | 0.275 [0.098, 0.453] | 0.002 | -0.225 [-0.263, -0.187] | 9.41×10^-32^ | 7.42 [1.676, 13.164] | 0.011 |
| 25 to <30 | 0.251 [0.23, 0.272] | 1.53×10^-122^ | 0.097 [0.092, 0.101] | < 5×10^-324^ | -0.404 [-1.078, 0.27] | 0.24 |
| 30 to <35 | 0.522 [0.496, 0.548] | < 5×10^-324^ | 0.147 [0.141, 0.152] | < 5×10^-324^ | -2.36 [-3.203, -1.518] | 4.00×10^-8^ |
| >35 | 0.949 [0.909, 0.988] | < 5×10^-324^ | 0.153 [0.144, 0.161] | 1.91×10^-275^ | -2.69 [-3.976, -1.403] | 4.19×10^-5^ |
| Continuous | 0.061 [0.059, 0.063] | < 5×10^-324^ | 0.012 [0.012, 0.013] | < 5×10^-324^ | -0.229 [-0.297, -0.162] | 2.05×10^-11^ |
| **Diabetes** | 0.269 [0.229, 0.309] | 3.63×10^-40^ | -0.098 [-0.107, -0.09] | 1.67×10^-116^ | -2.829 [-4.112, -1.546] | 1.55×10^-5^ |
| **Hypertension** | 0.23 [0.21, 0.251] | 5.37×10^-106^ | 0.004 [-6.5×10^-4^, 0.008] | 0.095 | 3.212 [2.546, 3.879] | 3.64×10^-21^ |
| **Hypercholesterolemia** | 0.146 [0.12, 0.172] | 9.33×10^-28^ | -0.018 [-0.024, -0.013] | 7.48×10^-11^ | -1.758 [-2.606, -0.911] | 4.76×10^-5^ |

^a^P-values from multivariable linear regression models adjusted for all other variables in this table. Unless specified otherwise, we adjusted for race/ethnicity, alcohol consumption, body mass index (continuous variable), diabetes, hypertension, and hypercholesterolemia. Except for continuous variable rows, the reference group for categorical variables were no mLOY, White, never drinker, 18.5 ≤ body mass index < 25, no diabetes, no hypertension, no hypercholesteroemia.
^b^The continuous variable reports the coefficient when mLRR was standardized.

**Table S19. Linear regression for lymphocyte, monocyte and neutrophil counts by mLOY status in more than 65 years old**

|  | Lymphocyte Count (×10^9^ cells/L) | | Monocyte Count (×10^9^ cells/L) | | Neutrophil Count (×10^9^ cells/L) | |
| --- | --- | --- | --- | --- | --- | --- |
|  | Estimate (95% CI) | *P*^a^ | Estimate (95% CI) | *P* | Estimate (95% CI) | *P* |
| **mLOY** |  |  |  |  |  |  |
| Categorical | 0.026 [0.016, 0.037] | 1.48×10^-6^ | 0.022 [0.019, 0.025] | 1.87×10^-45^ | 0.187 [0.167, 0.207] | 2.45×10^-78^ |
| Continuous^b^ | -0.002 [-0.006, 0.002] | 0.377 | 0.008 [0.007, 0.009] | 6.70×10^-34^ | 0.069 [0.061, 0.077] | 6.51×10^-63^ |
| **Race/Ethnicity** |  |  |  |  |  |  |
| Mixed | 0.12 [0.072, 0.168] | 1.10×10^-6^ | -0.019 [-0.033, -0.006] | 0.006 | -0.1 [-0.188, -0.012] | 0.026 |
| Asian | 0.333 [0.31, 0.356] | 8.38×10^-175^ | -0.004 [-0.011, 0.002] | 0.211 | -0.068 [-0.11, -0.026] | 0.002 |
| Black | 0.155 [0.126, 0.184] | 3.67×10^-26^ | -0.098 [-0.106, -0.09] | 5.36×10^-121^ | -1.305 [-1.357, -1.252] | < 5×10^-324^ |
| Other | 0.187 [0.15, 0.224] | 4.91×10^-23^ | -0.043 [-0.054, -0.033] | 9.19×10^-16^ | -0.359 [-0.427, -0.292] | 1.95×10^-25^ |
| **Smoking status** |  |  |  |  |  |  |
| Current smoker | 0.047 [0.039, 0.055] | 6.63×10^-30^ | 0.012 [0.01, 0.014] | 7.20×10^-24^ | 0.078 [0.063, 0.092] | 2.29×10^-24^ |
| Former smoker | 0.365 [0.354, 0.377] | < 5×10^-324^ | 0.066 [0.063, 0.07] | < 5×10^-324^ | 1.008 [0.987, 1.028] | < 5×10^-324^ |
| **Alcohol drinking** |  |  |  |  |  |  |
| Former drinker | -0.002 [-0.032, 0.029] | 0.918 | -0.009 [-0.018, -7.85×10^-4^] | 0.032 | 0.025 [-0.03, 0.08] | 0.368 |
| Occasional | 0.012 [-0.014, 0.038] | 0.374 | -0.001 [-0.009, 0.006] | 0.75 | 0.026 [-0.022, 0.074] | 0.286 |
| 1-3 drink/month | 0.011 [-0.015, 0.037] | 0.403 | -1.9×10^-4^ [-0.008, 0.007] | 0.96 | -0.028 [-0.075, 0.019] | 0.248 |
| 1-2 drink/week | 0.005 [-0.019, 0.03] | 0.661 | -0.005 [-0.012, 0.002] | 0.129 | -0.09 [-0.134, -0.046] | 5.95×10^-5^ |
| 3-4 drink/week | -0.013 [-0.038, 0.011] | 0.281 | -0.01 [-0.017, -0.003] | 0.007 | -0.197 [-0.241, -0.152] | 3.38×10^-18^ |
| Daily | -0.038 [-0.063, -0.014] | 0.002 | -0.001 [-0.008, 0.006] | 0.744 | -0.22 [-0.265, -0.175] | 5.30×10^-22^ |
| **Body mass index** |  |  |  |  |  |  |
| < 18.5 | -0.198 [-0.275, -0.122] | 3.39×10^-7^ | 0.005 [-0.017, 0.027] | 0.631 | 0.268 [0.128, 0.408] | 1.72×10^-4^ |
| 25 to <30 | 0.123 [0.114, 0.132] | 2.85×10^-160^ | 0.022 [0.02, 0.025] | 1.15×10^-65^ | 0.097 [0.08, 0.113] | 4.45×10^-31^ |
| 30 to <35 | 0.241 [0.23, 0.252] | < 5×10^-324^ | 0.053 [0.05, 0.056] | 7.60×10^-232^ | 0.207 [0.187, 0.228] | 5.64×10^-88^ |
| >35 | 0.317 [0.3, 0.334] | 1.51×10^-290^ | 0.084 [0.079, 0.089] | 3.00×10^-248^ | 0.514 [0.483, 0.545] | 1.06×10^-227^ |
| Continuous | 0.023 [0.022, 0.024] | < 5×10^-324^ | 0.006 [0.005, 0.006] | < 5×10^-324^ | 0.031 [0.029, 0.032] | 1.00×10^-294^ |
| **Diabetes** | 0.04 [0.023, 0.057] | 3.69×10^-6^ | 0.005 [-3.07×10^-4^, 0.009] | 0.066 | 0.205 [0.174, 0.236] | 5.17×10^-38^ |
| **Hypertension** | -0.009 [-0.018, -1.07×10^-4^] | 0.047 | 0.019 [0.017, 0.022] | 1.24×10^-50^ | 0.217 [0.201, 0.233] | 8.91×10^-152^ |
| **Hypercholesterolemia** | 0.043 [0.032, 0.054] | 1.06×10^-13^ | 0.014 [0.011, 0.017] | 4.66×10^-17^ | 0.086 [0.065, 0.106] | 2.78×10^-16^ |

^a^P-values from multivariable linear regression models adjusted for all other variables in this table. Unless specified otherwise, we adjusted for race/ethnicity, alcohol consumption, body mass index (continuous variable), diabetes, hypertension, and hypercholesterolemia. Except for continuous variable rows, the reference group for categorical variables were no mLOY, White, never drinker, 18.5 ≤ body mass index < 25, no diabetes, no hypertension, no hypercholesteroemia.
^b^The continuous variable reports the coefficient when mLRR was standardized.

|  | NLR^a^ | | | |
| --- | --- | --- | --- | --- |
|  | < 65 | | ≥ 65 | |
|  | Estimate (95% CI) | *P*^a^ | Estimate (95% CI) | *P* |
| **mLOY** |  |  |  |  |
| Categorical | 0.062 [0.028, 0.097] | 4.34×10^-4^ | 0.109 [0.092, 0.126] | 5.42×10^-36^ |
| Continuous^b^ | 0.05 [0.038, 0.063] | 3.79×10^-16^ | 0.043 [0.035, 0.05] | 2.95×10^-30^ |
| **Race/Ethnicity** |  |  |  |  |
| Mixed | -0.398 [-0.745, -0.051] | 0.024 | -0.222 [-0.302, -0.142] | 5.55×10^-8^ |
| Asian | -0.322 [-0.45, -0.193] | 8.82×10^-7^ | -0.424 [-0.462, -0.385] | 2.13×10^-104^ |
| Black | -1.048 [-1.247, -0.85] | 4.07×10^-25^ | -0.892 [-0.939, -0.844] | 1.81×10^-296^ |
| Other | -0.553 [-0.82, -0.286] | 4.95×10^-5^ | -0.435 [-0.497, -0.374] | 4.52×10^-44^ |
| **Smoking status** |  |  |  |  |
| Current smoker | 0.012 [-0.024, 0.048] | 0.514 | -0.011 [-0.025, 0.002] | 0.097 |
| Former smoker | 0.016 [-0.047, 0.079] | 0.615 | 0.074 [0.055, 0.092] | 5.23×10^-15^ |
| **Alcohol drinking** |  |  |  |  |
| Former drinker | 0.069 [-0.064, 0.201] | 0.31 | 0.018 [-0.032, 0.068] | 0.477 |
| Occasional | -0.044 [-0.158, 0.07] | 0.452 | 0.003 [-0.04, 0.047] | 0.882 |
| 1-3 drink/month | -0.021 [-0.137, 0.094] | 0.716 | -0.056 [-0.099, -0.013] | 0.01 |
| 1-2 drink/week | -0.099 [-0.203, 0.005] | 0.062 | -0.089 [-0.129, -0.049] | 1.30×10^-5^ |
| 3-4 drink/week | -0.129 [-0.233, -0.024] | 0.016 | -0.134 [-0.174, -0.094] | 6.58×10^-11^ |
| Daily | -0.154 [-0.258, -0.05] | 0.004 | -0.106 [-0.147, -0.066] | 2.85×10^-7^ |
| **Body mass index** |  |  |  |  |
| < 18.5 | -1.614 [-2.062, -1.166] | 1.75×10^-12^ | 0.521 [0.394, 0.649] | 1.06×10^-15^ |
| 25 to <30 | -1.835 [-2.282, -1.387] | 9.89×10^-16^ | -0.131 [-0.146, -0.116] | 8.94×10^-67^ |
| 30 to <35 | -1.921 [-2.37, -1.472] | 5.16×10^-17^ | -0.221 [-0.24, -0.202] | 2.37×10^-119^ |
| >35 | -1.937 [-2.392, -1.482] | 7.42×10^-17^ | -0.174 [-0.203, -0.146] | 3.80×10^-33^ |
| Continuous | -0.028 [-0.033, -0.024] | 1.37×10^-34^ | -0.016 [-0.018, -0.015] | 1.03×10^-103^ |
| **Diabetes** | 0.116 [0.057, 0.176] | 1.33×10^-4^ | 0.112 [0.084, 0.14] | 7.69×10^-15^ |
| **Hypertension** | 0.188 [0.153, 0.224] | 8.68×10^-25^ | 0.187 [0.173, 0.202] | 2.53×10^-140^ |
| **Hypercholesterolemia** | 0.003 [-0.036, 0.043] | 0.863 | 0.004 [-0.015, 0.022] | 0.68 |

**Table S20. Associations between neutrophil-lymphocyte ratio (NLR) and mLOY in subjects more or less than 65 years old**

^a^P-values from multivariable linear regression models adjusted for all other variables in this table. Unless specified otherwise, we adjusted for race/ethnicity, alcohol consumption, smoking, body mass index (continuous variable), diabetes, hypertension, and hypercholesterolemia. Except for continuous variable rows, the reference group for categorical variables were no mLOY, White, never smoker, never drinker, 18.5 ≤ body mass index < 25, no diabetes, no hypertension, no hypercholesteroemia.
^b^The continuous variable reports the coefficient when mLRR was standardized.

**Table S21. Associations between thrombocyte-lymphocyte ratio (TLR) and mLOY in subjects more or less than 65 years old**

|  | TLR^a^ | | | |
| --- | --- | --- | --- | --- |
|  | < 65 | | ≥ 65 | |
|  | Estimate (95% CI) | *P*^a^ | Estimate (95% CI) | *P* |
| **mLOY** |  |  |  |  |
| Categorical | 1.797 [0.261, 3.332] | 0.022 | 0.003 [-0.797, 0.803] | 0.994 |
| Continuous^b^ | 2.225 [1.688, 2.763] | 4.97×10^-16^ | 0.985 [0.642, 1.328] | 1.87×10^-8^ |
| **Race/Ethnicity** |  |  |  |  |
| Mixed | -10.09 [-25.444, 5.263] | 0.198 | -8.22 [-11.964, -4.477] | 1.68×10^-5^ |
| Asian | -11.112 [-16.786, -5.439] | 1.24×10^-4^ | -20.602 [-22.394, -18.811] | 2.21×10^-112^ |
| Black | -24.385 [-33.164, -15.607] | 5.23×10^-8^ | -22.357 [-24.576, -20.137] | 1.19×10^-86^ |
| Other | -21.107 [-32.928, -9.287] | 4.66×10^-4^ | -16.152 [-19.021, -13.283] | 2.65×10^-28^ |
| **Smoking status** |  |  |  |  |
| Current smoker | -0.323 [-1.93, 1.284] | 0.693 | -1.824 [-2.452, -1.197] | 1.22×10^-8^ |
| Former smoker | -15.839 [-18.628, -13.051] | 9.49×10^-29^ | -19.135 [-20.001, -18.269] | < 5×10^-324^ |
| **Alcohol drinking** |  |  |  |  |
| Former drinker | 1.795 [-4.072, 7.661] | 0.549 | -1.374 [-3.708, 0.96] | 0.249 |
| Occasional | -2.495 [-7.55, 2.56] | 0.333 | -1.96 [-4.008, 0.089] | 0.061 |
| 1-3 drink/month | -0.259 [-5.385, 4.867] | 0.921 | -2.753 [-4.762, -0.744] | 0.007 |
| 1-2 drink/week | -2.632 [-7.242, 1.979] | 0.263 | -2.898 [-4.772, -1.023] | 0.002 |
| 3-4 drink/week | -1.088 [-5.707, 3.532] | 0.644 | -2.977 [-4.86, -1.094] | 0.002 |
| Daily | -0.509 [-5.105, 4.088] | 0.828 | -1.435 [-3.334, 0.463] | 0.138 |
| **Body mass index** |  |  |  |  |
| < 18.5 | -40.845 [-59.719, -21.971] | 2.22×10^-5^ | 24.88 [18.849, 30.911] | 6.22×10^-16^ |
| 25 to <30 | -57.443 [-76.289, -38.598] | 2.33×10^-9^ | -11.608 [-12.313, -10.902] | 2.62×10^-227^ |
| 30 to <35 | -66.257 [-85.157, -47.357] | 6.45×10^-12^ | -21.285 [-22.167, -20.404] | < 5×10^-324^ |
| >35 | -73.083 [-92.231, -53.934] | 7.55×10^-14^ | -27.191 [-28.538, -25.845] | < 5×10^-324^ |
| Continuous | -2.495 [-2.694, -2.295] | 2.33×10^-131^ | -2.002 [-2.071, -1.933] | < 5×10^-324^ |
| **Diabetes** | -1.021 [-3.661, 1.619] | 0.448 | -2.422 [-3.745, -1.099] | 3.33×10^-4^ |
| **Hypertension** | 4.088 [2.499, 5.678] | 4.65×10^-7^ | 3.075 [2.395, 3.756] | 8.29×10^-19^ |
| **Hypercholesterolemia** | -4.574 [-6.314, -2.833] | 2.62×10^-7^ | -5.098 [-5.966, -4.23] | 1.15×10^-30^ |

^a^P-values from multivariable linear regression models adjusted for all other variables in this table. Unless specified otherwise, we adjusted for race/ethnicity, smoking, alcohol consumption, body mass index (continuous variable), diabetes, hypertension, and hypercholesterolemia. Except for continuous variable rows, the reference group for categorical variables were no mLOY, White, never smoker, never drinker, 18.5 ≤ body mass index < 25, no diabetes, no hypertension, no hypercholesteroemia.
^b^The continuous variable reports the coefficient when mLRR was standardized.

**Table S22. Association between polygenic risk scores, mLRR and blood cell indices**

|  | Male mLRR | | Male PRS^a^ | | Female PRS | |
| --- | --- | --- | --- | --- | --- | --- |
|  | Estimate | Pr(Pr(>\|t\|) ) | Estimate | Pr(Pr(>\|t\|) ) | Estimate | Pr(Pr(>\|t\|) ) |
| Leukocyte | 0.057 [0.049, 0.065] | 5.43×10^-44^ | 0.045 [0.036, 0.053] | 4.12×10^-27^ | 0.035 [0.028, 0.043] | 7.1×10^-21^ |
| Erythrocyte | -0.009 [-0.01, -0.007] | 2.21×10^-23^ | 0.004 [0.002, 0.005] | 1.51×10^-5^ | 0.008 [0.007, 0.009] | 6.74×10^-30^ |
| Thrombocyte | 2.278 [2.022, 2.534] | 5.47×10^-68^ | 1.553 [1.311, 1.795] | 3.27×10^-36^ | 1.643 [1.397, 1.888] | 2.15×10^-39^ |
| Lymphocyte | -0.002 [-0.006, 0.002] | 0.291 | 0.001 [-0.002, 0.004] | 0.557 | -0.002 [-0.005, 6.86×10^-4^] | 0.128 |
| Monocyte | 0.005 [0.004, 0.006] | 1.92×10^-25^ | 0.005 [0.004, 0.006] | 3.36×10^-21^ | 0.003 [0.002, 0.004] | 4.81×10^-11^ |
| Neutrophil | 0.054 [0.048, 0.06] | 9.15×10^-64^ | 0.039 [0.032, 0.045] | 9.65×10^-34^ | 0.034 [0.028, 0.039] | 2.82×10^-31^ |
| NLR | 0.038 [0.032, 0.044] | 1.65×10^-33^ | 0.023 [0.017, 0.029] | 1.88×10^-14^ | 0.019 [0.015, 0.024] | 3.78×10^-17^ |
| TLR | 1.695 [1.414, 1.976] | 2.73×10^-32^ | 1.051 [0.779, 1.324] | 4.00×10^-14^ | 0.991 [0.691, 1.291] | 1.01×10^-10^ |

^a^PRS: polygenic risk scores based on 156 SNPs associated with mLOY^1^

**Table S23. Reciprocal Mendelian Randomization**

| SNP^a^ | Outcome | Inverse-variance weighted | |  | Egger | |
| --- | --- | --- | --- | --- | --- | --- |
|  |  | Estimate (95% CI) | *P* |  | Estimate (95% CI) | *P* |
| mLOY | leukocyte | 0.132 [0.078, 0.186] | 1.67×10^-6^ |  | 0.263 [0.172, 0.354] | 1.64×10^-8^ |
| leukocyte | mLOY | 0.778 [0.635, 0.922] | 2.01×10^-26^ |  | 0.825 [0.644, 1.006] | < 5×10^-324^ |
| mLOY | erythrocyte | -0.009 [-0.023, 0.005] | 0.215 |  | -0.028 [-0.052, -0.004] | 0.024 |
| erythrocyte^b^ | mLOY | -0.053 [-0.07, -0.036] | 4.99×10^-10^ |  | -0.08 [-0.103, -0.058] | 5.36×10^-12^ |
| mLOY | thrombocyte | 3.59 [1.838, 5.342] | 5.91×10^-5^ |  | 5.446 [2.403, 8.488] | 4.51×10^-4^ |
| thrombocyte^c^ | mLOY | 22.561 [19.694, 25.429] | 1.18×10^-53^ |  | 16.874 [13.171, 20.577] | < 5×10^-324^ |

^a^See supplementary file SNPs_used_in_MR.xlsx for SNPs used in MR

**Table S24. The potential mediation effect of mLOY on the association between age and blood cell counts in leukocyte, erythrocyte, and thrombocyte**

|  | Leukocyte Count (×10^9^ cells/L) | | Erythrocyte Count (×10^12^ cells/L) | | Thrombocyte Count (×10^9^ cells/L) | |
| --- | --- | --- | --- | --- | --- | --- |
|  | Estimate (95% CI) | *P*^a^ | Estimate (95% CI) | *P* | Estimate (95% CI) | *P* |
| ACME^b^ | 2.38×10^-4^ [1.57×10^-4^, 3.18×10^-4^] | < 5×10^-324^ | -4.49×10^-5^ [-5.76×10^-5^, -3.21×10^-5^] | < 5×10^-324^ | 0.007 [0.005, 0.009] | < 5×10^-324^ |
| ADE^c^ | 0.017 [0.016, 0.018] | < 5×10^-324^ | -0.005 [-0.005, -0.005] | < 5×10^-324^ | -0.604 [-0.629, -0.573] | < 5×10^-324^ |
| TE^d^ | 0.018 [0.017, 0.018] | < 5×10^-324^ | -0.005 [-0.005, -0.005] | < 5×10^-324^ | -0.597 [-0.623, -0.565] | < 5×10^-324^ |
| PM^e^ | 0.013 [0.009, 0.019] | < 5×10^-324^ | 0.009 [0.006, 0.011] | < 5×10^-324^ | -0.012 [-0.015, -0.008] | < 5×10^-324^ |

^a^Besides 2 level mLOY as the mediator, continuous age as the exposure, and blood cell counts as outcome, all models were adjusted for 25 level smoking, ethnicity, alcohol consumption, diabetes, hypertension, cholesterolemia, and continuous BMI.

^b^Average causal mediation effect (ACME)

^c^Average direct effect (ADE)

^d^Total effect (TE)

^e^Proportion mediated (PM)

**Table S25. The potential mediation effect of mLOY on the association between age and blood cell counts in lymphocyte, monocyte, and neutrophil**

|  | Lymphocyte Count (×10^9^ cells/L) | | | Monocyte Count (×10^9^ cells/L) | | | | Neutrophil Count (×10^9^ cells/L) | | | |  |
| --- | --- | --- | --- | --- | --- | --- | --- | --- | --- | --- | --- | --- |
|  | Estimate (95% CI) | *P* | | Estimate (95% CI) | | *P* | | Estimate (95% CI) | | *P* | |  |
| ACME | 1.86×10^-5^ [-3.48×10^-5^, 1.03×10^-4^] | | 0.560 | | 1.79×10^-5^ [1.25×10^-5^, 2.49×10^-5^] | | < 5×10^-324^ | | 2.06×10^-4^ [1.63×10^-4^, 2.52×10^-4^] | | < 5×10^-324^ | |
| ADE | -5.76×10^-4^ [-9.49×10^-4^, -2.07×10^-4^] | | < 5×10^-324^ | | 0.003 [0.002, 0.003] | | < 5×10^-324^ | | 0.015 [0.014, 0.016] | | < 5×10^-324^ | |
| TE | -5.58×10^-4^ [-9.21×10^-4^, -1.75×10^-4^] | | < 5×10^-324^ | | 0.003 [0.002, 0.003] | | < 5×10^-324^ | | 0.015 [0.015, 0.016] | | < 5×10^-324^ | |
| PM | -0.033 [-0.353, 0.085] | | 0.560 | | 0.007 [0.005, 0.01] | | < 5×10^-324^ | | 0.013 [0.011, 0.016] | | < 5×10^-324^ | |

^a^Besides 2 level mLOY as the mediator, continuous age as the exposure, and blood cell counts as outcome, all models were adjusted for 25 level smoking, ethnicity, alcohol consumption, diabetes, hypertension, cholesterolemia, and continuous BMI.

^b^Average causal mediation effect (ACME)

^c^Average direct effect (ADE)

^d^Total effect (TE)

^e^Proportion mediated (PM)

**Table S26. The potential mediation effect of mLOY on the association between smoking and blood cell counts in leukocyte, erythrocyte, and thrombocyte**

|  |  | Leukocyte Count (×10^9^ cells/L) | | Erythrocyte Count (×10^12^ cells/L) | | Thrombocyte Count (×10^9^ cells/L) | | |
| --- | --- | --- | --- | --- | --- | --- | --- | --- |
|  |  | Estimate (95% CI) | *P*^a^ | Estimate (95% CI) | *P* | | Estimate (95% CI) | *P* |
| ACME^b^ | Current^f^ | 0.005 [0.003, 0.007] | < 5×10^-324^ | -9.17×10^-4^ [-0.001, -6.08×10^-4^] | < 5×10^-324^ | | 0.158 [0.101, 0.201] | < 5×10^-324^ |
|  | Former | 8.95×10^-4^ [3.98×10^-4^, 0.001] | < 5×10^-324^ | -1.29×10^-4^ [-2.34×10^-4^, -8.35×10^-5^] | < 5×10^-324^ | | 0.025 [0.014, 0.038] | < 5×10^-324^ |
| ADE^c^ | Current | 1.504 [1.477, 1.533] | < 5×10^-324^ | -0.057 [-0.062, -0.052] | < 5×10^-324^ | | 9.477 [8.695, 10.267] | < 5×10^-324^ |
|  | Former | 0.148 [0.131, 0.163] | < 5×10^-324^ | -0.031 [-0.034, -0.027] | < 5×10^-324^ | | 4.075 [3.559, 4.612] | < 5×10^-324^ |
| TE^d^ | Current | 1.509 [1.481, 1.538] | < 5×10^-324^ | -0.058 [-0.063, -0.053] | < 5×10^-324^ | | 9.635 [8.838, 10.423] | < 5×10^-324^ |
|  | Former | 0.149 [0.132, 0.164] | < 5×10^-324^ | -0.031 [-0.034, -0.027] | < 5×10^-324^ | | 4.099 [3.59, 4.632] | < 5×10^-324^ |
| PM^e^ | Current | 0.003 [0.002, 0.005] | < 5×10^-324^ | 0.016 [0.01, 0.021] | < 5×10^-324^ | | 0.016 [0.01, 0.021] | < 5×10^-324^ |
|  | Former | 0.006 [0.003, 0.009] | < 5×10^-324^ | 0.004 [0.003, 0.008] | < 5×10^-324^ | | 0.006 [0.003, 0.009] | < 5×10^-324^ |

^a^Besides 2 level mLOY as the mediator, continuous age as the exposure, and blood cell counts as outcome, all models were adjusted for age, age squared, ethnicity, alcohol consumption, diabetes, hypertension, cholesterolemia, and continuous BMI.

^b^Average causal mediation effect (ACME)

^c^Average direct effect (ADE)

^d^Total effect (TE)

^e^Proportion mediated (PM)

^f^Compared to never smokers

**Table S27. The potential mediation effect of mLOY on the association between smoking and blood cell counts in lymphocyte, monocyte, and neutrophil**

|  |  | Lymphocyte Count (×10^9^ cells/L) | | Monocyte Count (×10^9^ cells/L) | | Neutrophil Count (×10^9^ cells/L) | |
| --- | --- | --- | --- | --- | --- | --- | --- |
|  |  | Estimate (95% CI) | *P*^a^ | Estimate (95% CI) | *P* | Estimate (95% CI) | *P* |
| ACME^b^ | Current^f^ | 4.88×10^-4^ [-5.46×10^-4^, 0.002] | 0.494 | 3.64×10^-4^ [2.29×10^-4^, 5.04×10^-4^] | < 5×10^-324^ | 0.004 [0.003, 0.005] | < 5×10^-324^ |
|  | Former | 8.02×10^-5^ [-1.09×10^-4^, 3.93×10^-4^] | 0.504 | 6.56×10^-5^ [2.97×10^-5^, 9.67×10^-5^] | < 5×10^-324^ | 8×10^-4^ [3.79×10^-4^, 0.001] | < 5×10^-324^ |
| ADE^c^ | Current | 0.363 [0.352, 0.373] | < 5×10^-324^ | 0.07 [0.067, 0.074] | < 5×10^-324^ | 1.022 [1, 1.046] | < 5×10^-324^ |
|  | Former | 0.047 [0.039, 0.054] | < 5×10^-324^ | 0.011 [0.009, 0.013] | < 5×10^-324^ | 0.081 [0.069, 0.095] | < 5×10^-324^ |
| TE^d^ | Current | 0.363 [0.352, 0.374] | < 5×10^-324^ | 0.07 [0.067, 0.074] | < 5×10^-324^ | 1.027 [1.004, 1.051] | < 5×10^-324^ |
|  | Former | 0.047 [0.039, 0.055] | < 5×10^-324^ | 0.011 [0.009, 0.013] | < 5×10^-324^ | 0.082 [0.07, 0.095] | < 5×10^-324^ |
| PM^e^ | Current | 0.001 [-0.001, 0.006] | 0.494 | 0.005 [0.003, 0.007] | < 5×10^-324^ | 0.004 [0.003, 0.005] | < 5×10^-324^ |
|  | Former | 0.002 [-0.002, 0.008] | 0.504 | 0.006 [0.003, 0.009] | < 5×10^-324^ | 0.01 [0.004, 0.013] | < 5×10^-324^ |

^a^Besides 2 level mLOY as the mediator, continuous age as the exposure, and blood cell counts as outcome, all models were adjusted for age, age squared, ethnicity, alcohol consumption, diabetes, hypertension, cholesterolemia, and continuous BMI.

^b^Average causal mediation effect (ACME)

^c^Average direct effect (ADE)

^d^Total effect (TE)

^e^Proportion mediated (PM)

^f^Compared to never smokers

**Figure S1. mLOY prevalence by decile.** Black line: overall. Red line: never smokers. Blue line: former smokers. Green line: current smokers.


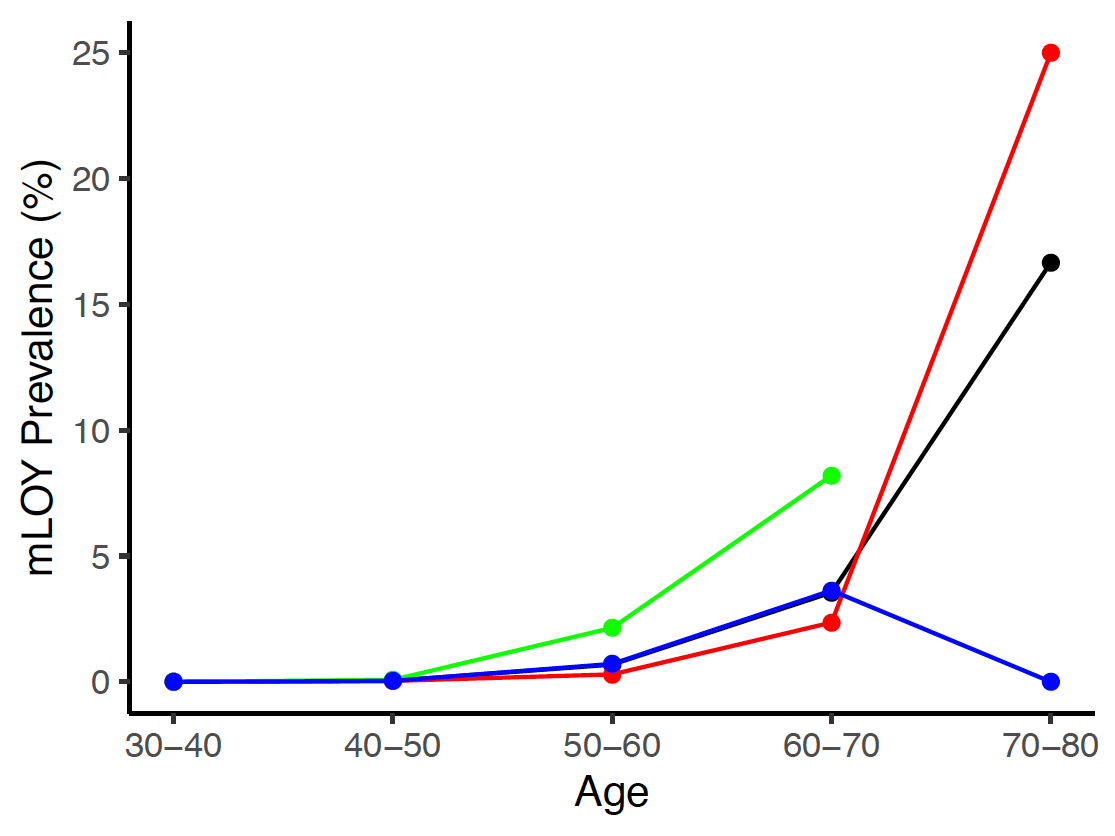


**Figure S2.** **Relative impact of selected risk factors associated with lymphocyte, monocyte, and neutrophil counts**. Multivariable linear regression models adjusted for age, age squared, race/ethnicity, smoking, alcohol consumption, body mass index (continuous variable), diabetes, hypertension, and hypercholesterolemia. The reference group for categorical variables were no mLOY, White, never smoker, never drinker, 18.5 ≤ body mass index < 25, no diabetes, no hypertension, no hypercholesteroemia.


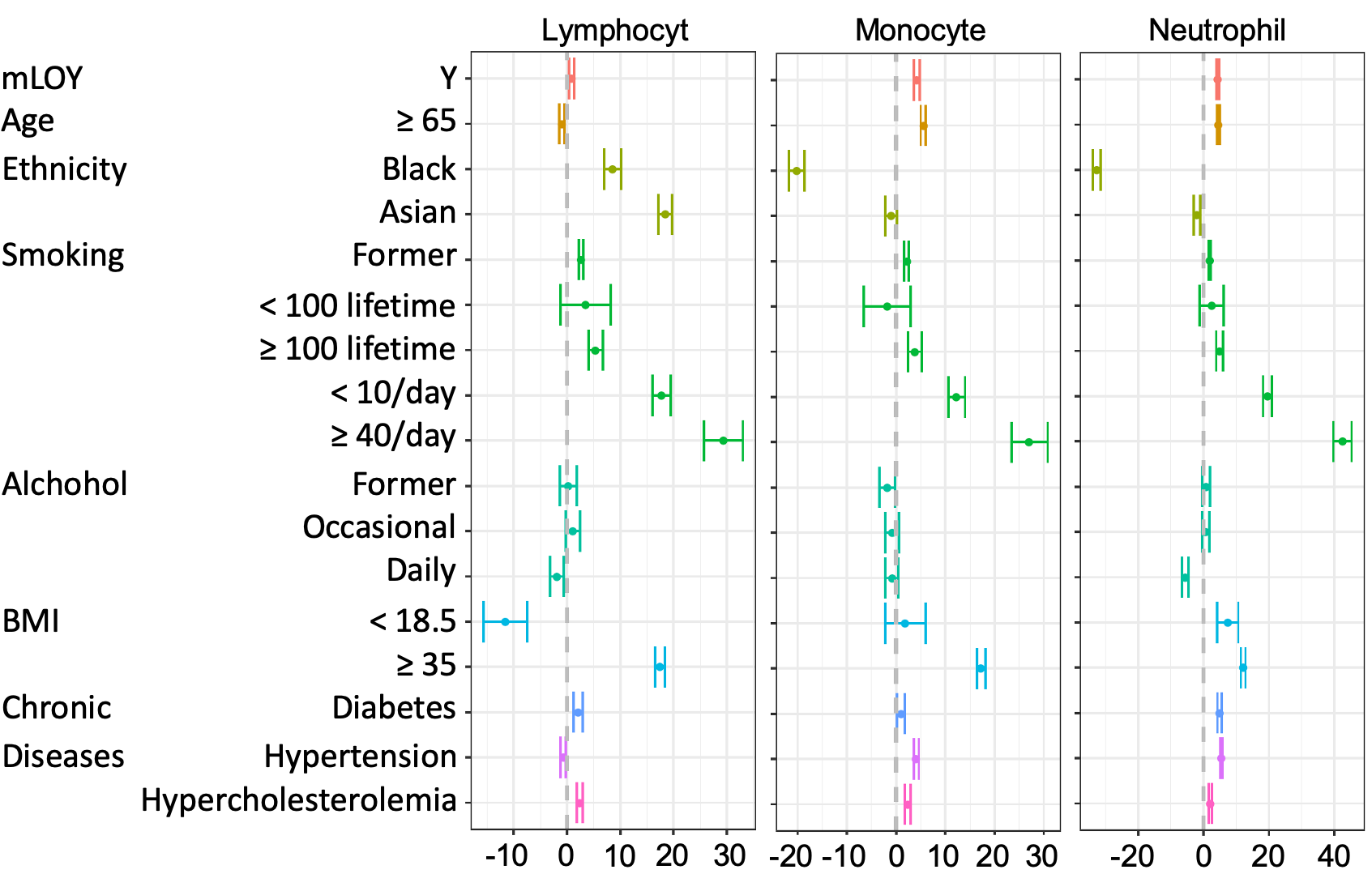


**Fig S3. mLRR and predicted lymphocyte count stratified by smoking status.** Red line: never smokers. Blue line: former smokers. Green line: current smokers. Upper left: leukocyte count. Upper middle: erythrocyte count. Upper right: thrombocyte count. Lower left: lymphocyte count. Lower middle: monocyte count. Lower right: neutrophil count. Multivariable linear regression models adjusted for age, age squared, race/ethnicity, smoking, alcohol consumption, body mass index (continuous variable), diabetes, hypertension, and hypercholesterolemia. The reference group for categorical variables were no mLOY, White, never smoker, never drinker, 18.5 ≤ body mass index < 25, no diabetes, no hypertension, no hypercholesteroemia.

**Figure S4.** Causal effects estimate by Mendelian randomization among mLOY, blood cell counts, and associated SNPs. Top left: leukocyte count and mLOY-associated SNPs. Top middle: erythrocyte count and mLOY-associated SNPs. Top right: thrombocyte count and mLOY-associated SNPs. Lower left: mLOY and leukocyte count-associated SNPs. Lower middle: mLOY and erythrocyte count-associated SNPs. Lower right: mLOY and thrombocyte count-associated SNPs.
